# An analysis-ready and quality controlled resource for pediatric brain white-matter research

**DOI:** 10.1038/s41597-022-01695-7

**Published:** 2022-10-12

**Authors:** Adam Richie-Halford, Matthew Cieslak, Lei Ai, Sendy Caffarra, Sydney Covitz, Alexandre R. Franco, Iliana I. Karipidis, John Kruper, Michael Milham, Bárbara Avelar-Pereira, Ethan Roy, Valerie J. Sydnor, Jason D. Yeatman, The Fibr Community Science Consortium, Nicholas J. Abbott, John A. E. Anderson, B. Gagana, MaryLena Bleile, Peter S. Bloomfield, Vince Bottom, Josiane Bourque, Rory Boyle, Julia K. Brynildsen, Navona Calarco, Jaime J. Castrellon, Natasha Chaku, Bosi Chen, Sidhant Chopra, Emily B. J. Coffey, Nigel Colenbier, Daniel J. Cox, James Elliott Crippen, Jacob J. Crouse, Szabolcs David, Benjamin De Leener, Gwyneth Delap, Zhi-De Deng, Jules Roger Dugre, Anders Eklund, Kirsten Ellis, Arielle Ered, Harry Farmer, Joshua Faskowitz, Jody E. Finch, Guillaume Flandin, Matthew W. Flounders, Leon Fonville, Summer B. Frandsen, Dea Garic, Patricia Garrido-Vásquez, Gabriel Gonzalez-Escamilla, Shannon E. Grogans, Mareike Grotheer, David C. Gruskin, Guido I. Guberman, Edda Briana Haggerty, Younghee Hahn, Elizabeth H. Hall, Jamie L. Hanson, Yann Harel, Bruno Hebling Vieira, Meike D. Hettwer, Harriet Hobday, Corey Horien, Fan Huang, Zeeshan M. Huque, Anthony R. James, Isabella Kahhale, Sarah L. H. Kamhout, Arielle S. Keller, Harmandeep Singh Khera, Gregory Kiar, Peter Alexander Kirk, Simon H. Kohl, Stephanie A. Korenic, Cole Korponay, Alyssa K. Kozlowski, Nevena Kraljevic, Alberto Lazari, Mackenzie J. Leavitt, Zhaolong Li, Giulia Liberati, Elizabeth S. Lorenc, Annabelle Julina Lossin, Leon D. Lotter, David M. Lydon-Staley, Christopher R. Madan, Neville Magielse, Hilary A. Marusak, Julien Mayor, Amanda L. McGowan, Kahini P. Mehta, Steven Lee Meisler, Cleanthis Michael, Mackenzie E. Mitchell, Simon Morand-Beaulieu, Benjamin T. Newman, Jared A. Nielsen, Shane M. O’Mara, Amar Ojha, Adam Omary, Evren Özarslan, Linden Parkes, Madeline Peterson, Adam Robert Pines, Claudia Pisanu, Ryan R. Rich, Matthew D. Sacchet, Ashish K. Sahoo, Amjad Samara, Farah Sayed, Jonathan Thore Schneider, Lindsay S. Shaffer, Ekaterina Shatalina, Sara A. Sims, Skyler Sinclair, Jae W. Song, Griffin Stockton Hogrogian, Christian K. Tamnes, Ursula A. Tooley, Vaibhav Tripathi, Hamid B. Turker, Sofie Louise Valk, Matthew B. Wall, Cheryl K. Walther, Yuchao Wang, Bertil Wegmann, Thomas Welton, Alex I. Wiesman, Andrew G. Wiesman, Mark Wiesman, Drew E. Winters, Ruiyi Yuan, Sadie J. Zacharek, Chris Zajner, Ilya Zakharov, Gianpaolo Zammarchi, Dale Zhou, Benjamin Zimmerman, Kurt Zoner, Theodore D. Satterthwaite, Ariel Rokem

**Affiliations:** 1 Stanford University, Division of Developmental and Behavioral Pediatrics, Stanford, California 94305 USA; 2 Stanford University, Graduate School of Education, Stanford, California 94305 USA; 3 Lifespan Informatics and Neuroimaging Center (PennLINC), Department of Psychiatry, Perelman School of Medicine, University of Pennsylvania, Philadelphia, Pennsylvania 19104 USA; 4 Penn/CHOP Lifespan Brain Institute, Perelman School of Medicine, Children’s Hospital of Philadelphia Research Institute, Philadelphia, Pennsylvania 19104 USA; 5 Department of Psychiatry, Perelman School of Medicine, University of Pennsylvania, Philadelphia, Pennsylvania 19104 USA; 6 Child Mind Institute, Center for the Developing Brain, New York City, New York 10022 USA; 7 University of Modena and Reggio Emilia, Department of Biomedical, Metabolic and Neural Sciences, 41125 Modena, Italy; 8 Nathan Kline Institute for Psychiatric Research, Center for Biomedical Imaging and Neuromodulation, Orangeburg, New York 10962 USA; 9 Stanford University, Department of Psychiatry and Behavioral Sciences, School of Medicine, Stanford, California 94305 USA; 10 University of Zurich, Department of Child and Adolescent Psychiatry and Psychotherapy, University Hospital of Psychiatry Zurich, Zurich, 8032 Switzerland; 11 Neuroscience Center Zurich, University of Zurich and ETH Zurich, Zurich, 8057 Switzerland; 12 University of Washington, Department of Psychology, Seattle, Washington 98195 USA; 13 University of Washington, eScience Institute, Seattle, Washington 98195 USA; 14 Old Dominion University, Norfolk, VA 23529 USA; 15 Carleton University, Northfield, MN 55057 USA; 16 Southern Methodist University, Dallas, TX 75275 USA; 17 University of California-Davis, Davis, CA 95616 USA; 18 University of Pennsylvania, Philadelphia, PA 19104 USA; 19 Harvard University, Cambridge, MA 2138 USA; 20 University of Toronto, Toronto, ON M5T 2S8 Canada; 21 Duke University, Durham, NC 27708 USA; 22 University of Michigan-Ann Arbor, Ann Arbor, MI 48109 USA; 23 San Diego State University, San Diego, CA 92182 USA; 24 University of California-San Diego, La Jolla, CA 92093 USA; 25 Concordia University, Montréal, QC H4B 1R6 Canada; 26 Katholieke Universiteit Leuven, 3000 Leuven, Belgium; 27 University of Manchester, Manchester, M13 9PL United Kingdom; 28 University of Sydney, Camperdown, NSW 2006 Australia; 29 University Medical Center Utrecht, 3584 CX Utrecht, Netherlands; 30 Polytechnique Montreal, Montréal, QC H3T 1J4 Canada; 31 University of Rochester, Rochester, NY 14627 USA; 32 National Institute of Mental Health, Bethesda, MD 20892 USA; 33 Centre de Recherche de l’Institut Universitaire en Santé Mentale de Montréal, Montréal, QC H1N 3M5 Canada; 34 Linköping university, 581 83 Linköping, Sweden; 35 Monash University, Clayton, VIC 3800 Australia; 36 Temple University, Philadelphia, PA 19122 USA; 37 University of Greenwich, London, SE10 9LS United Kingdom; 38 Indiana University-Bloomington, Bloomington, IN 47405 USA; 39 Georgia State University, Atlanta, GA 30303 USA; 40 University College London, London, WC1E 6BT United Kingdom; 41 Imperial College London, London, SW7 2BX United Kingdom; 42 Brigham and Women’s Hospital, Boston, MA 02115 USA; 43 University of North Carolina at Chapel Hill, Chapel Hill, NC 27599 USA; 44 University of Concepción, Concepción, Bio Bio Chile; 45 Universitätsmedizin der Johannes Gutenberg-Universität Mainz, 55131 Mainz, Germany; 46 University of Maryland-College Park, College Park, MD 20742 USA; 47 Philipps-Universität Marburg, Marburg, 35037 Germany; 48 Columbia University, New York, NY 10027 USA; 49 McGill University, Montreal, Quebec H3A 0G4 Canada; 50 University of Pittsburgh, Pittsburgh, PA 15260 USA; 51 University of Montréal, Montreal, Quebec, H3T 1J4 Canada; 52 Universidade de São Paulo, Ribeirão Preto, Brazil; 53 Heinrich-Heine University Dusseldorf, 40225 Düsseldorf, Germany; 54 Yale University, New Haven, CT 6520 USA; 55 University of Chicago, Chicago, IL 60637 USA; 56 Brigham Young University, Provo, UT 84602 USA; 57 Child Mind Institute, New York, NY 10022 USA; 58 Institute of Neurosciences and Medicine, Forschungszentrum Jülich, 52425 Jülich, Germany; 59 McLean Hospital, Belmont, MA 02478 USA; 60 University of Oxford, Oxford, OX1 2JD United Kingdom; 61 Auburn University, Auburn, AL 36849 USA; 62 Washington University in St Louis, Saint Louis, MO 63130 USA; 63 Université catholique de Louvain, 1348 Ottignies-Louvain-la-Neuve, Belgium; 64 University of Texas at Austin, Austin, TX 78705 USA; 65 University of Nottingham, Nottingham, NG7 2RD United Kingdom; 66 Wayne State University, Detroit, MI 48202 USA; 67 University of Oslo, 0315 Oslo, Norway; 68 University of Michigan, Ann Arbor, MI 48109 USA; 69 University of Virginia, Charlottesville, VA 22903 USA; 70 Trinity College Dublin, Dublin, 2 Ireland; 71 Linköping University, 581 83 Linköping, Sweden; 72 Stanford University, Stanford, CA 94305 USA; 73 University of Cagliari, 09124 Cagliari, CA Italy; 74 Massachusetts General Hospital, Harvard Medical School, Boston, USA; 75 University of Florida, Gainesville, FL 32611 USA; 76 George Mason University, Fairfax, VA 22030 USA; 77 University of Alabama at Birmingham, Birmingham, AL 35294 USA; 78 Boston University, Boston, MA 2215 USA; 79 Cornell University, Ithaca, NY 14853 USA; 80 Max Planck Institute for Human Cognitive and Brain Sciences, 04103 Leipzig, Germany; 81 National Neuroscience Institute, Singapore, 308433 Singapore; 82 University of Colorado School of Medicine, Aurora, CO 80045 USA; 83 Massachusetts Institute of Technology, Cambridge, MA 02139 USA; 84 Western Unviersity, London, ON N6A 3K7 Canada; 85 Psychological Institute of Russian Academy of Education, Moscow, 129366 Russia; 86 University of Illinois Urbana-Champaign, Champaign, IL 61820 USA

**Keywords:** Cognitive neuroscience, Computational neuroscience

## Abstract

We created a set of resources to enable research based on openly-available diffusion MRI (dMRI) data from the Healthy Brain Network (HBN) study. First, we curated the HBN dMRI data (N = 2747) into the Brain Imaging Data Structure and preprocessed it according to best-practices, including denoising and correcting for motion effects, susceptibility-related distortions, and eddy currents. Preprocessed, analysis-ready data was made openly available. Data quality plays a key role in the analysis of dMRI. To optimize QC and scale it to this large dataset, we trained a neural network through the combination of a small data subset scored by experts and a larger set scored by community scientists. The network performs QC highly concordant with that of experts on a held out set (ROC-AUC = 0.947). A further analysis of the neural network demonstrates that it relies on image features with relevance to QC. Altogether, this work both delivers resources to advance transdiagnostic research in brain connectivity and pediatric mental health, and establishes a novel paradigm for automated QC of large datasets.

## Abstract (summary)

**Table:** 

Measurement(s)	brain white matter
Technology Type(s)	Diffusion Weighted Magnetic Resonance Imaging
Sample Characteristic - Organism	Homo sapiens
Sample Characteristic - Location	contiguous United States of America

## Background & Summary

Childhood and adolescence are characterized by rapid dynamic changes to human brain structure and function^1^. This period of development is also a time during which the symptoms of many mental health disorders emerge^2^. Understanding how individual differences in brain development relate to the onset and progression of psychopathology inevitably requires large datasets^3,4^. The Healthy Brain Network (HBN) is a landmark pediatric mental health study that is designed to eventually include MRI images along with detailed clinical and cognitive phenotyping from over 5000 New York City area children and adolescents^5,6^. The HBN dataset takes a trans-diagnostic approach and provides a broad range of phenotypic and brain imaging data for each individual. One of the brain imaging measurements acquired is diffusion MRI (dMRI), which is the dominant technology for inferring the physical properties of white matter^7^. The dMRI data is openly available in its raw form through the Functional Connectomes Project and the International Neuroimaging Data-Sharing Initiative (FCP-INDI), spurring collaboration on open and reproducible science^8^.

However, this raw, publicly available data requires extensive processing and quality assurance before it can be fruitfully analyzed. The most immediate contribution of the present work is a large openly-available analysis-ready dMRI data resource derived from the HBN dataset^9^. In the past decade, projects such as the Human Connectome Project (HCP)^10^, UK Biobank^11^, ABCD^12^, and CamCAN^13,14^, as well as FCP-INDI, have ushered a culture of data sharing in open big-data human neuroscience. The adoption and reuse of these datasets reduces or eliminates the data collection burden on downstream researchers. Some projects, such as the HCP^15^, also provide preprocessed derivatives, further reducing researchers’ burden and extending the benefits of data-sharing from data collection to preprocessing and secondary analysis. Following the example of the HCP, the present study provides analysis-ready dMRI derivatives from HBN. This avoids duplication of and heterogeneity across the preprocessing effort, while also ensuring a high standard of data quality for HBN researchers.

The analysis of a large, multi-site dMRI dataset must take into account the inevitable variability in scanning parameters across scanning sessions. Critical preprocessing steps, such as susceptibility distortion correction^16^ require additional MRI acquisitions besides dMRI and accurate metadata accompanying each image. A session missing an acquisition or important metadata can either be processed to the extent its available data allows or excluded entirely. In addition, the quality of preprocessed data is heavily affected by differences in acquisition parameters^17^ and by differences in preprocessing steps. Here we address these problems by meticulously curating the HBN data according to the Brain Imaging Data Specification (BIDS)^18^ and processing the data using the *QSIPrep*^19^ BIDS App^20^. *QSIPrep* automatically builds and executes benchmarked workflows that adhere to best practices in the field given the available BIDS data. The results include automated data quality metrics, visual reports and a description of the processing steps automatically chosen to process each session.

This preprocessing requires a costly compute infrastructure and is both time-consuming and error-prone. Requiring researchers to process dMRI data on their own introduces both a practical barrier to access and an extra source of heterogeneity into the data, devaluing its scientific utility. We provide the preprocessed data as a transparent and open resource, thereby reducing barriers to data access and allowing researchers to spend more of their time answering questions in brain development and psychopathology rather than recapitulating preprocessing.

In addition to requiring extensive preprocessing, dMRI data must be thoroughly checked for quality. dMRI measurements are susceptible to a variety of artifacts that affect the quality of the signals and the ability to make accurate inferences from them. In small studies, with few participants, it is common to thoroughly examine the data from every participant as part of a quality control (QC) process. However, expert examination is time consuming and is prohibitive in large datasets such as HBN. This difficulty could be ameliorated through the automation of QC. Given their success in other visual recognition tasks, machine learning and computer vision methods, such as convolutional deep artificial neural networks or “deep learning”^21^, are promising avenues for automation of QC. However, one of the challenges of these new methods is that they require a large training dataset to attain accurate performance. In previous work, we demonstrated that deep learning can accurately emulate expert QC of T1-weighted (T1w) anatomical brain images^22^. To obtain a large enough training dataset of T1w images in our prior study, we deployed a community science tool that collected quality control scores of parts of the dataset from volunteers through a web application. The scores were then calibrated using a gold standard expert-scored subset of these images. A deep learning neural network was trained on the calibrated and aggregated score, resulting in very high concordance with expert ratings on a separate test dataset. We termed this approach “hybrid QC”, because it combined information from experts with information from community scientists to create a scalable machine learning algorithm that can be applied to future data collection.

However, the hybrid QC proof-of-concept left lingering questions about its applicability to other datasets because it was trained on a single-site, single-modality dataset. Here, we expand the hybrid-QC approach to a large multi-site dMRI dataset. Moreover, one of the common critiques of deep learning is that it can learn irrelevant features of the data and does not provide information that is transparent enough to interpret^23–25^. To confirm that the hybrid-QC deep learning algorithm uses meaningful features of the diffusion-weighted images (DWI) to perform accurate QC, we used machine learning interpretation methods that pry open the “black box” of the neural network, thereby highlighting the features that lead to a specific QC score^26,27^.

Taken together, the combination of curated BIDS data, preprocessed images, and quality control scores generated by the deep learning algorithm provides researchers with a rich and accessible data resource. Making MRI derivatives accessible not only reduces the burden of processing large datasets for research groups with limited resources^28^, but also aids research performed by clinicians who are interested in brain-behavior relationships but may be lacking the technical training to process large-scale dMRI data. We anticipate that these HBN Preprocessed Open Diffusion Derivatives (HBN-POD2) will accelerate translational research on both normal and abnormal brain development.

## Methods

**Fig. 1:**
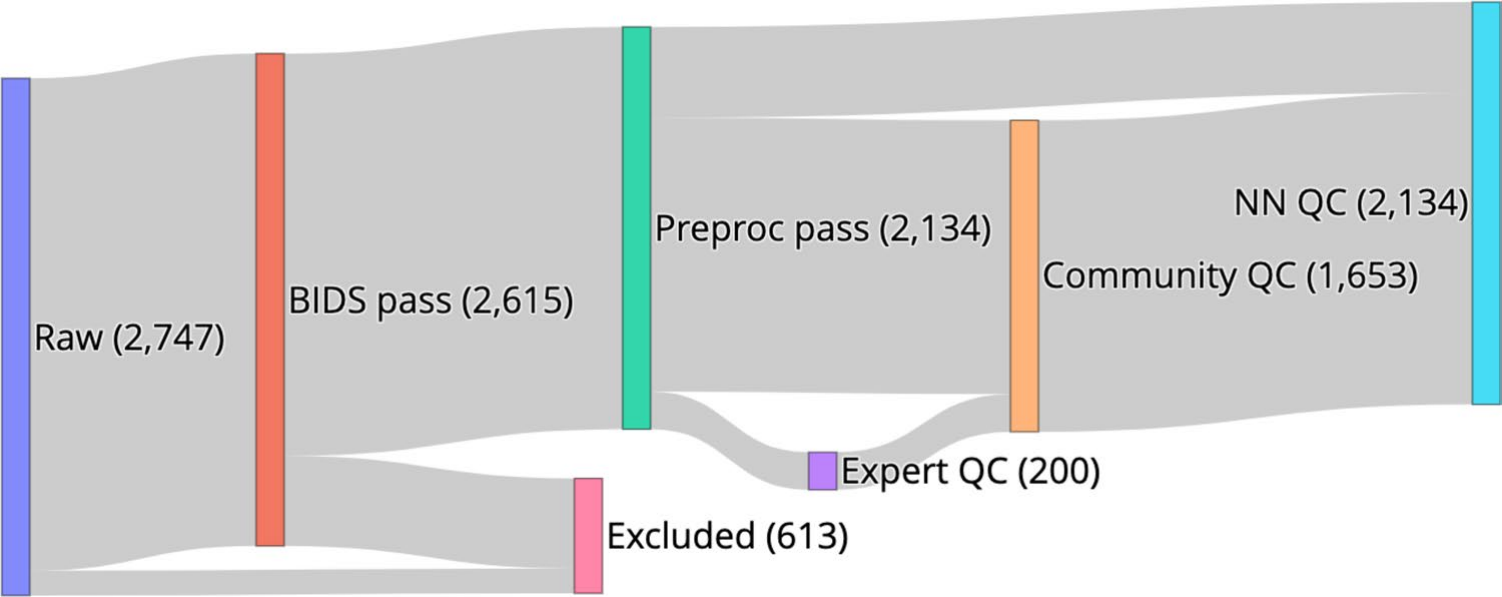
HBN-POD2 data provenance: Imaging data for 2,747 participants, aged 5–21 years and collected at four sites in the New York City area, was made available through the Functional Connectomes Project and the International Neuroimaging Data-Sharing Initiative (FCP-INDI). These data were curated for compliance to the BIDS specification^18^ and availability of imaging metadata in json format. 2615 participants met this specification. Imaging data was preprocessed using *QSIPrep*^19^ to group, distortion correct, motion correct, denoise, coregister and resample MRI scans. Of the BIDS curated participants, 2,134 passed this step, with the majority of failures coming from participants with missing dMRI scans. Expert raters assigned QC scores to 200 of these participants, creating a “gold standard” QC subset (Fig. 2). Community raters then assigned binary QC ratings to a superset of the gold standard containing 1,653 participants. An image classification algorithm was trained on a combination of automated quality metrics from *QSIPrep* and community scientist reviews to “extend” the expert ratings to the community science subset (Fig. 4). Finally, a deep learning QC model was trained on the community science subset to assign QC scores to the entire dataset and to future releases from HBN (Fig. 7). The HBN-POD2 dataset, including QC ratings, is openly available through FCP-INDI.

The aims of this data resource were fourfold (i) curate the HBN MRI data into a fully BIDS-compliant MRI dataset, (ii) perform state-of-the-art diffusion MRI (dMRI) preprocessing using *QSIPrep*, (iii) assign QC scores to each participant, and (iv) provide unrestricted public release to the outputs from each of these steps. We started with MRI data from 2,747 HBN participants available through FCP-INDI, curating these data for compliance with the Brain Imaging Data Structure (BIDS) specification^18^. We preprocessed the structural MRI (sMRI) and diffusion MRI (dMRI) data using *QSIPrep*. Participants that could not be curated to comply with the BIDS standard or that did not have dMRI data were excluded, resulting in 2,134 participants with preprocessed, BIDS-compliant dMRI data (Fig. 1).

### Inputs

Inputs for this study consisted of MRI data from releases 1–9 of the Healthy Brain Network pediatric mental health study^5,6^, containing dMRI data from 2,747 participants aged 5–21 years. These data were measured using a 1.5 T Siemens mobile scanner on Staten Island (SI, *N* = 300) and three fixed 3 T Siemens MRI scanners at sites in the New York area: Rutgers University Brain Imaging Center (RU, *N* = 873), the CitiGroup Cornell Brain Imaging Center (CBIC, *N* = 887), and the City University of New York Advanced Science Research Center (CUNY, *N* = 74), where numbers in parentheses represent participant counts in HBN-POD2. Site CBIC has two different acquisition types: one which shares its pulse sequence with sites RU and CUNY and another (with only 19 participants), which better matches the ABCD study diffusion protocol^29^. Informed consent was obtained from each participant aged 18 or older. For participants younger than 18, written consent was obtained from their legal guardians and written assent was obtained from the participant. Voxel resolution was 1.8 mm × 1.8 mm × 1.8 mm with 64 non-colinear directions measured for each two degrees of diffusion weighting: *b* = 1500 s/mm^2^ and *b* = 3000 s/mm^2^ for the ABCD-harmonized sequence and *b* = 1000 s/mm^2^ and *b* = 2000 s/mm^2^ for the others. Figure 10 depicts the age distribution of study participants by sex for each of these scan sites as well as pairwise distributions for the automated quality metrics that are described in the next sections.

### BIDS curation

We curated the imaging metadata for 2,615 of the 2,747 currently available HBN participants. Using dcm2bids and custom scripts, we conformed the data to the Brain Imaging Data Structure (BIDS)^18^ specification. The BIDS-curated dataset is available on FCP-INDI and can be accessed via AWS S3 at s3://fcp-indi/data/Projects/HBN/BIDS_curated/.

After conforming the data to BIDS, we used the “Curation of BIDS” (CuBIDS) package^30^ to identify unique combinations, or “variants” of imaging parameters in the curated dMRI and fieldmap acquisitions. CuBIDS is a Python-based software package that provides a sanity-preserving workflow to help users reproducibly parse, validate, curate, and understand heterogeneous BIDS imaging datasets. CuBIDS includes a robust implementation of the BIDS Validator that scales to large samples and incorporates DataLad^31^, a distributed data management system, to ensure reproducibility and provenance tracking throughout the curation process. CuBIDS tools also employ agglomerative clustering to identify variants of imaging parameters. Each session was grouped according to metadata parameters that affect the dMRI signal (PhaseEncodingDirection, EchoTime, VoxelSize, FlipAngle, PhasePartialFourier, NumberOfVolumes, Fieldmap availability). We identified a total of 20 unique DWI acquisitions across HBN-POD2, where about 5% of acquisitions were different from the most common DWI acquisition at their site.

### Preprocessing

We performed dMRI preprocessing on 2615 participants, using *QSIPrep*^19^ 0.12.1, which is based on *Nipype* 1.5.1^32,33^, RRID:SCR_002502. *QSIPrep* is a robust and scalable pipeline to group, distortion correct, motion correct, denoise, coregister and resample MRI scans. In total, 417 participants failed this preprocessing step, largely due to missing dMRI files. In keeping with the BIDS specification, the preprocessed dataset is available as a derivative dataset within the BIDS-curated dataset and can be access on AWS S3 at s3://fcp-indi/data/Projects/HBN/BIDS_curated/derivatives/qsiprep/. *QSIPrep* fosters reproducibility by automatically generating thorough methods boilerplate text for later use in scientific publications, which we use for the remainder of this subsection to document each preprocessing step.

- *Anatomical data preprocessing*. All T1-weighted (T1w) images found for each participant were corrected for intensity non-uniformity (INU) using N4BiasFieldCorrection^34^ (ANTs 2.3.1). If a single T1w was found, it was used as the T1w-reference throughout the workflow. If multiple T1w images were found, a T1w-reference map was computed after registration of the T1w images (after INU-correction) using mri_robust_template^35^ (FreeSurfer 6.0.1). The T1-weighted (T1w) image was corrected for intensity non-uniformity (INU) using N4BiasFieldCorrection^34^ (ANTs 2.3.1), and used as T1w-reference throughout the workflow. The T1w-reference was then skull-stripped using antsBrainExtraction.sh (ANTs 2.3.1), using OASIS as target template. Spatial normalization to the ICBM 152 Nonlinear Asymmetrical template version 2009c (RRID:SCR_008796)^36^ was performed through nonlinear registration with antsRegistration (ANTs 2.3.1, RRID:SCR_004757)^37^, using brain-extracted versions of both T1w volume and template. Brain tissue segmentation of cerebrospinal fluid (CSF), white-matter (WM) and gray-matter (GM) was performed on the brain-extracted T1w using FAST (FSL 6.0.3:b862cdd5, RRID:SCR_002823)^38^.
- *Diffusion data preprocessing*. Any images with a *b*-value less than 100 s/mm^2^ were treated as a *b* = 0 image. MP-PCA denoising as implemented in MRtrix3’s dwidenoise^39^ was applied with a 5-voxel window. After MP-PCA, B1 field inhomogeneity was corrected using dwibiascorrect from MRtrix3 with the N4 algorithm^34^. After B1 bias correction, the mean intensity of the DWI series was adjusted so all the mean intensity of the *b* = 0 images matched across each separate DWI scanning sequence. FSL’s (version 6.0.3:b862cdd5) eddy was used for head motion correction and eddy current correction^40^. Eddy was configured with a *q*-space smoothing factor of 10, a total of 5 iterations, and 1000 voxels used to estimate hyperparameters. A linear first level model and a linear second level model were used to characterize eddy current-related spatial distortion. *q*-space coordinates were forcefully assigned to shells. Field offset was attempted to be separated from participant movement. Shells were aligned post-eddy. Eddy’s outlier replacement was run^41^. Data were grouped by slice, only including values from slices determined to contain at least 250 intracerebral voxels. Groups deviating by more than four standard deviations from the prediction had their data replaced with imputed values. Data was collected with reversed phase-encode blips, resulting in pairs of images with distortions going in opposite directions. Here, *b* = 0 reference images with reversed phase encoding directions were used along with an equal number of *b* = 0 images extracted from the DWI scans. From these pairs the susceptibility-induced off-resonance field was estimated using a method similar to that described in^42^. The fieldmaps were ultimately incorporated into the Eddy current and head motion correction interpolation. Final interpolation was performed using the jac method. Several confounding time-series were calculated based on the *preprocessed DWI*: framewise displacement (FD) using the implementation in *Nipype* following the definitions by^43^. The DWI time-series were resampled to ACPC, and their corresponding gradient directions were rotated accordingly to generate a preprocessed DWI run in ACPC space

Many internal operations of *QSIPrep* use *Nilearn* 0.6.2^44^, RRID:SCR_001362 and *DIPY*^45^. For more details of the pipeline, see the section corresponding to workflows in QSIPrep’s documentation.

### Cloud-based distributed preprocessing

The containerization of *QSIPrep* provided a consistent preprocessing pipeline for each participant but the number of participants made serial processing of each participant prohibitive on a single machine. We used *cloudknot*, a previously developed cloud-computing library^46^ to parallelize the preprocessing over individual participants on spot instances in the Amazon Web Services Batch service. *Cloudknot* takes as input a user-defined Python function and creates the necessary AWS infrastructure to map that function onto a range of inputs, in this case, the participant IDs. Using *cloudknot* and AWS Batch Spot Instances, the preprocessing cost less than $1.00 per participant.

### Quality control

To QC all available HBN dMRI data, we adopted a hybrid QC approach that combines expert rating, community science, and deep learning, drawing on the success of a previous application in assessing the quality of HBN’s structural T1w MRI data^22^. This method (i) starts with dMRI expert raters labelling a small subset of participants, the “gold standard” dataset (ii) amplifies these labels using a community science web application to extend expert ratings to a much larger subset of the data, the community science subset and (iii) trains a deep learning model on the community science subset to predict expert decisions on the entire dataset.

#### Expert quality control

The expert QC “gold standard” subset was created by randomly selecting 200 participants from the preprocessed dataset, sampled such that the proportional site distribution in the gold standard subset matched that of the preprocessed dataset.

We then developed *dmriprep-viewer*, a dMRI data viewer and QC rating web application to display *QSIPrep* outputs and collect expert ratings^47^. The viewer ingests *QSIPrep* outputs and generates a browser-based interface for expert QC. It provides a study overview displaying the distributions of *QSIPrep*’s automated data quality metrics (described at https://qsiprep.readthedocs.io/en/latest/preprocessing.html#quality-control-data). Each datum on the study overview page is interactively linked to a participant-level QC page that provides an interactive version of *QSIPrep*’s visual reports (described at https://qsiprep.readthedocs.io/en/latest/preprocessing.html#visual-reports). The viewer allows users to assign a rating of −2 (definitely fail), −1 (probably fail), 0 (not sure), 1 (probably pass), or 2 (definitely pass) to a participant. To standardize rater expectations before rating, expert raters watched a tutorial video (available on YouTube at https://youtu.be/SQ0v-O-e5b8 and in the OSF project), which demonstrated data for which each of these ratings was appropriate. Six of the co-authors, who are all dMRI experts, rated the gold standard subset using extensive visual examination of each participant’s dMRI data, including the preprocessed dMRI time series, a plot of motion parameters throughout the dMRI scan, and full 3D volumes depicting (i) the brain mask and *b* = 0 to T1w registration and (ii) a directionally encoded color fractional anisotropy (DEC-FA) image laid over the *b* = 0 volume. See Fig. 3 for an example of the *dmriprep-viewer* interface.

**Fig. 2:**
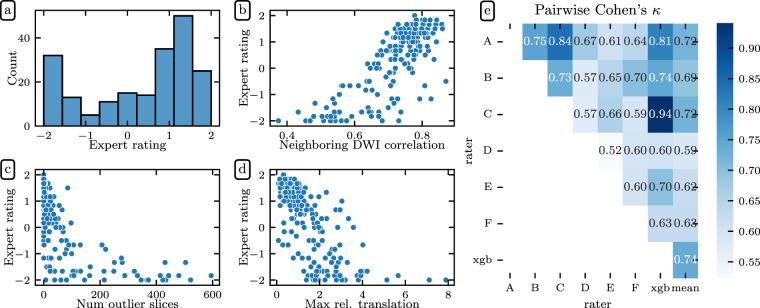
Expert QC results: Six dMRI experts rated a subset of 200 participants. Experts agreed with *QSIPrep*’s automated QC metrics. Here we show the distribution of mean expert QC ratings **(a)** and associations between the mean expert QC rating and the *QSIPrep* metrics **(b)** neighboring diffusion-weighted imaging (DWI) correlation^17^, **(c)** maximum relative translation, and **(d)** number of outlier slices. As expected, neighboring DWI correlation is directly correlated with expert rating while the other two metrics are inversely correlated with expert rating. **(e)** Experts agreed with each other. Here we show the pairwise Cohen’s *κ* measure of inter-rater reliability (see text for ICC calculations). The XGB model has an inter-rater reliability (quantified here as Cohen’s *κ*) that is indistinguishable from the other raters.

The distribution of scores given by the experts demonstrates that the gold standard dataset included a range of data quality (Fig. 2a). Mean expert ratings correlated with the three *QSIPrep* automated QC metrics that were most informative for the XGB model described in the next section: neighboring diffusion-weighted imaging (DWI) correlation^17^ (Fig. 2b), maximum relative translation (Fig. 2c), and number of outlier slices (Fig. 2d). The neighboring DWI correlation characterizes the pairwise spatial correlation between pairs of DWI volumes that sample neighboring points in *q*-space. Since lower values indicate reduced data quality, it is reassuring that the neighboring DWI correlation correlated directly with expert ratings (Pearson CC: 0.797). Conversely, high relative translation and a high number of motion outlier slices reflect poor data quality and these metrics were inversely related to mean expert rating (Pearson CC: −0.692 and Pearson CC: −0.695, respectively).

In addition to agreeing qualitatively with *QSIPrep*’s automated QC metrics on average, the expert raters also tended to agree with each other (Fig. 2e). We assessed inter-rater reliability (IRR) using the pairwise Cohen’s *κ*^48^, computed using the *scikit-learn*^49^ cohen_kappa_score function with quadratic weights. The pairwise *κ* exceeded 0.52 in all cases, with a mean value of 0.648. In addition to the pairwise Cohen’s *κ*, we also computed the intra-class correlation (ICC)^50^ as a measure of IRR, using the *pingouin* statistical package^51^ intraclass_corr function. ICC3k is the appropriate variant of the ICC to use when a fixed set of *k* raters each code an identical set of participants, as is the case here. ICC3k for inter-rater reliability among the experts was 0.930 (95% CI: [0.91, 0.94]), which is qualitatively considered an “excellent” level of IRR^52^. The high IRR provides confidence that the average of the expert ratings for each image in the gold standard is an appropriate target to use for training a machine learning model that predicts the expert scores.

#### Community scientist quality control

**Fig. 3:**
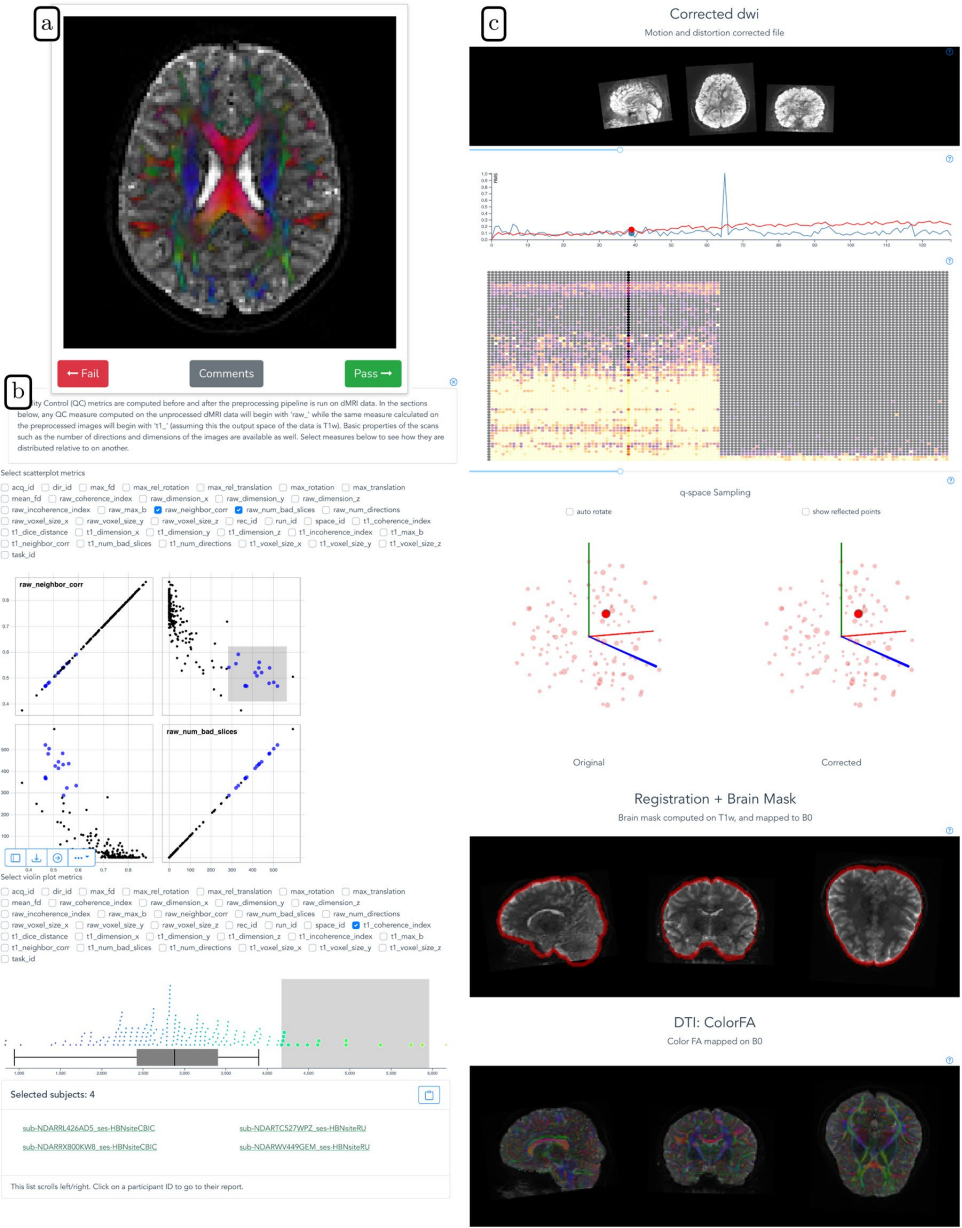
HBN-POD2 quality control instruments: (**a**) The user interface for community science QC app *Fibr*. After a tutorial, users are asked to give binary pass/fail ratings to each subject’s DEC-FA image. The intuitive swipe or click interface allows community scientists to review more images than is practical for expert reviewers. Expert reviewers use the more advanced *dmriprep-viewer* interface, where they can **(b)** view the distribution of data quality metrics for the entire study using interactive scatterplots and violin plots, and **(c)** inspect individual participants’ preprocessing results, including corrected dMRI images, frame displacement, q-space sampling distributions, registration information, and a DTI model.

Although the expert raters achieved high IRR and yielded intuitive associations with *QSIPrep*’s automated QC metrics, generating expert QC labels for the entire HBN-POD2 dataset would be prohibitively time consuming. To assess the image quality of the remaining participants, we deployed *Fibr* (https://fibr.dev), a community science web application in which users assigned binary pass/fail labels assessing the quality of horizontal slice DEC-FA images overlaid on the *b* = 0 image (see Fig. 3 for an example). Specifically, after a brief tutorial, *Fibr* users saw individual slices or an animated sequence of ten slices taken from the entire DEC-FA volume that the expert raters saw. The *Fibr* users, therefore, saw only a subset of the imaging data that the dMRI experts had access to for a given participant, but they saw data from many more participants. In total, 374 community scientists provided 587,778 ratings for a mean of >50 ratings per slice (or >200 ratings per participant) from 1,653 participants. Of the community scientists, 145 raters provided >3,000 ratings each and are included in the *Fibr* Community Science Consortium as co-authors on this paper^53^.

We created quality control web applications for both community raters and expert raters. These apps are publicly accessible at https://fibr.dev and at http://www.nipreps.org/dmriprep-viewer/, for the community science instrument and the expert rating instrument, respectively. We encourage readers to try these web applications on their own but have included screenshots and a summary of the interfaces in Fig. 3.

**Fig. 4:**
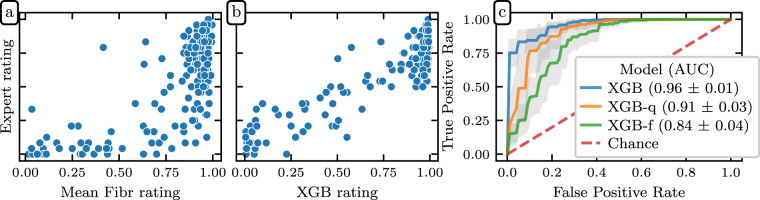
Community science predictions of the expert ratings: Scatter plots showing the relationship between mean expert rating and both mean *Fibr* rating **(a)** and XGB prediction **(b)**. *Fibr* raters overestimated the quality of images compared to expert raters. But the XGB prediction compensated for this by incorporating automated QC metrics and weighting more valuable *Fibr* raters. **(c)** ROC curves for the XGB, XGB-q, and XGB-f models. Translucent bands represent one standard deviation from the mean of the cross-validation splits.

There are three issues to account for when comparing *Fibr* and expert QC ratings. First, the unadjusted *Fibr* ratings were overly optimistic; i.e., on average, community scientists were not as conservative as the expert raters (Fig. 4a). Second, different community scientists provide data of differing accuracy. That is, they were less consistent across different views of the same image, and/or were less consistent with expert ratings for the same data. This means that data from some *Fibr* raters was more informative than others. Third, important information about data quality was provided in the *QSIPrep* data quality metrics, which were not available to *Fibr* raters. To account for rater variability and take advantage of the information provided by *QSIPrep*, we trained gradient boosted decision trees^54^ to predict expert scores, scaled to the range [0,1] and binarized with a 0.5 threshold, based on a combination of community science ratings and 31 automated *QSIPrep* data quality metrics. One can think of the gradient boosting model as assigning more weight to *Fibr* raters who reliably agree with the expert raters, thereby resolving the aforesaid issues with community rater accuracy. We refer to this gradient boosting model as XGB.

All gradient boosting models were implemented as binary classifiers using the XGBoost library^55^. The targets for these classifiers were the mean expert ratings in the gold standard dataset, rescaled to the range [0, 1] and binarized with a threshold of 0.5. Using repeated stratified K-fold cross-validation, with three splits and two repeats, we evaluated the models’ performance in predicting the gold standard ratings. In each fold, the best model hyperparameters were chosen using the scikit-optimize^56^ BayesSearchCV class. Since each split resulted in a different XGB model and we required a single QC score to train the deep learning model, we combined the models from each cross-validation split using a voting classifier, computing a weighted averaged of the predicted probability of passing from each model, weighted by its out-of-sample ROC-AUC. This was implemented using scikit-learn’s VotingClassifier class.

To clarify the contributions of the automated QC metrics and the community science raters, we trained two additional gradient boosting models: (i) one trained only on the automated *QSIPrep* data quality metrics, which we call XGB-q and (ii) one trained on only the *Fibr* ratings, which we call XGB-f. XGB-f may be viewed as a data-driven weighting of community scientists’ ratings, while XGB-q may be viewed as a generalization of data quality metric exclusion criteria. XGB, combining information from both *Fibr* ratings and *QSIPrep* data quality metrics attained a cross-validated area under the receiver operating curve (ROC-AUC) of 0.96 ± 0.01 on the “gold standard,” where the ± indicates the standard deviation of scores from repeated *k*-fold cross-validation (Fig. 4b). In contrast, XGB-q attained an ROC-AUC of 0.91 ± 0.03 and XGB-f achieved an ROC-AUC of 0.84 ± 0.04. The enhanced performance of XGB-q over XGB-f shows that community scientists alone are not as accurate as automated data quality metrics are at predicting expert ratings. And yet, the increased performance of XGB over XGB-q demonstrates that there is additional image quality information to be gained by incorporating community scientist input.

We used SHapley Additive exPlanations (SHAP) to measure the global feature importance of the automated quality metrics in the gradient boosting models. SHAP is a method to explain individual predictions based on game theoretically optimal Shapley values^57^. To estimate global feature importance for the XGB and XGB-q models, we used the shap library’s TreeExplainer^58^ and averaged the absolute Shapley value per feature across each individual prediction. Tables 1 and 2 list the *QSIPrep* automated QC metric features in order of decreasing mean absolute shap value for the XGB and XGB-q models, respectively. We chose the top three metrics from Table 1 to plot metric distributions in Fig. 10 and correlations with the expert QC results in Fig. 2.

**Table 1:** XGB mean absolute shap values.

feature	mean abs shap
raw_neighbor_corr	0.666429
max_rel_translation	0.348662
raw_num_bad_slices	0.288937
t1_neighbor_corr	0.282198
raw_incoherence_index	0.229733
raw_coherence_index	0.162103
max_rel_rotation	0.118963
mean_fd	0.116457
max_fd	0.099359
max_rotation	0.078774
t1_coherence_index	0.035553
t1_dice_distance	0.034510
max_translation	0.032323
t1_incoherence_index	0.030225
raw_voxel_size_x	0.000000
raw_voxel_size_y	0.000000
raw_voxel_size_z	0.000000
raw_num_directions	0.000000
raw_max_b	0.000000
raw_dimension_y	0.000000
raw_dimension_z	0.000000
t1_voxel_size_x	0.000000
t1_dimension_x	0.000000
t1_dimension_y	0.000000
t1_dimension_z	0.000000
t1_voxel_size_y	0.000000
t1_voxel_size_z	0.000000
t1_max_b	0.000000
t1_num_bad_slices	0.000000
t1_num_directions	0.000000
raw_dimension_x	0.000000

**Table 2:** XGB-q mean absolute shap values.

feature	mean abs shap
raw_neighbor_corr	0.767536
raw_incoherence_index	0.453897
raw_num_bad_slices	0.430422
t1_coherence_index	0.382218
max_rel_translation	0.363052
raw_coherence_index	0.320438
t1_neighbor_corr	0.250948
t1_dice_distance	0.248104
t1_incoherence_index	0.242348
max_rel_rotation	0.135590
mean_fd	0.128642
max_translation	0.120815
max_fd	0.119739
max_rotation	0.101209
t1_num_bad_slices	0.007075
raw_dimension_y	0.000000
raw_dimension_z	0.000000
raw_voxel_size_x	0.000000
raw_voxel_size_y	0.000000
raw_voxel_size_z	0.000000
raw_max_b	0.000000
t1_voxel_size_x	0.000000
raw_num_directions	0.000000
t1_dimension_x	0.000000
t1_dimension_y	0.000000
t1_dimension_z	0.000000
t1_voxel_size_y	0.000000
t1_voxel_size_z	0.000000
t1_max_b	0.000000
t1_num_directions	0.000000
raw_dimension_x	0.000000

As a way of evaluating the quality of the XGB predictions, consider the fact that the average Cohen’s *κ* between XGB and the expert raters was 0.74, which is higher than the average Cohen’s *κ* between any of the other raters and their human peers (Fig. 2). This is not surprising, given that the XGB model was fit to optimize this match, but further demonstrates the goodness of fit of this model.

Nevertheless, this provides confidence in using the XGB scores in the next step of analysis, where we treat the XGB model as an additional coder and extend XGB ratings to participants without *Fibr* ratings. In this case, when a subset of participants is coded by multiple raters and the reliability of their ratings is meant to generalize to other participants rated by only one coder, the single-measure ICC3, as opposed to ICC3k, should be used. When adding XGB to the existing expert raters as a seventh expert, we achieved *ICC*3 = 0.709(95% *CI*: [0.66, 0.75]). The high ICC3 value after inclusion of the XGB model justifies using the XGB scores as the target for training an image-based deep learning network.

#### Automated quality control labelling through deep learning

While the XGB “rater” does a good job of extending QC ratings to the entire community science subset, this approach requires *Fibr* scores; without community science *Fibr* scores, only the less accurate XGB-q prediction can be employed. Consequently, a new, fully automated QC approach is needed that can be readily applied to future data releases from HBN.

We therefore trained deep convolutional neural networks to predict binarized XGB ratings directly from *QSIPrep* outputs. We modified an existing 3D convolutional neural network (CNN) architecture^59^–previously applied to the ImageCLEF Tuberculosis Severity Assessment 2019 benchmark^60^–to accept multichannel input generated from the preprocessed dMRI: the *b* = 0 reference diffusion image, each of the three cardinal axis components of the DEC-FA image, and, optionally, automated QC metrics from *QSIPrep*. We trained these networks on XGB scores and validated it against the gold standard expert-scored dataset. We refer to the convolutional neural network model trained only on imaging data as CNN-i and the model that incorporates automated QC metrics as CNN-i + q.

**Fig. 5:**
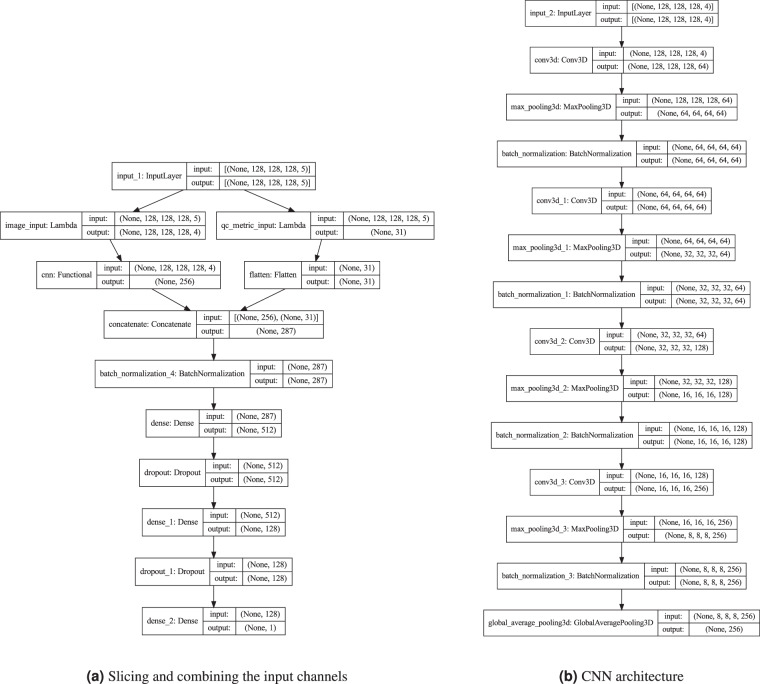
Deep learning model architecture: (**a**) The CNN-i + q model accepts multichannel input that combined four imaging channels with a fifth channel containing 31 *QSIPrep* automated data quality metrics. The imaging channels are separated from the data quality channel using Lambda layers. The imaging channels are passed through a CNN **(b)**, the output of which is concatenated with the data quality metrics, batch normalized and passed through two fully-connected (FC) layers, with rectified linear unit (ReLu) activation functions and with 512 and 128 units respectively. Each FC layer is followed by a dropout layer which drops 40% of the input units. The final layer contains a single unit with a sigmoid activation function and outputs the probability of passing QC. **(b)** The CNN portion of the model passes the imaging input through four convolutional blocks. Each block consists of a 3D convolutional layer with a kernel size of 3 and a ReLu activation, a 3D max pooling layer with a pool size of 2, and a batch normalization layer with Tensorflow’s default parameters. The number of filters in the convolutional layers in each block are 64, 64, 128, and 256 respectively. The output of the final block is passed through a 3D global average pooling layer with Tensorflow’s default parameters.

Both the CNN-i and CNN-i + q models were implemented in Tensorflow 2^61^ using the Keras module^62^. The image processing part of the model architecture was identical for both models: a modification of an existing 3D CNN^59^ previously applied to assess tuberculosis severity^60^. It accepts a 3D volume as input with four channels: (i) the *b* = 0 reference volume, (ii) DEC-FA in the *x*-direction, (iii) DEC-FA in the *y*-direction and (iv) DEC-FA in the *z*-direction. The *QSIPrep*’s automated QC metrics were included as an additional fifth channel. The CNN-i + q model architecture is summarized in Fig. 5. Upon input, the CNN-i + q model extracts the imaging channels and passes them through the CNN architecture. The remaining data quality metrics channel is flattened and passed “around” the CNN architecture and concatenated with the output of the convolutional layers. This concatenated output is then passed through a fully-connected layer to produce a single output, the probability of passing QC. This architecture has 1,438,783 trainable parameters.

**Fig. 6:**
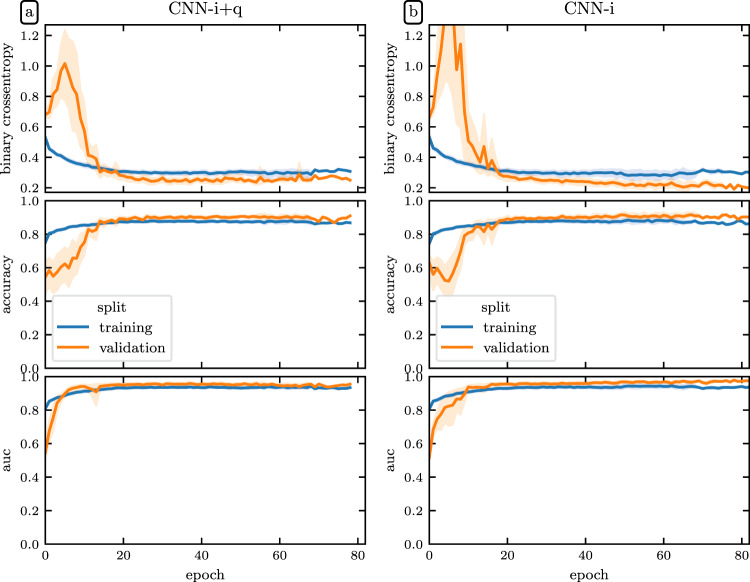
Deep learning model loss curves: The binary cross-entropy loss (top), accuracy (middle), and ROC-AUC (bottom) for **(a)** the CNN-i + q model and **(a)** the CNN-i model. Model performance typically plateaued after twenty epochs but was allowed continue until meeting the early stopping criterion. The error bands represent a bootstrapped 95% confidence interval.

To estimate the variability in model training, we trained ten separate models using different training and validation splits of the data. The gold standard dataset was not included in any of these splits and was reserved for reporting final model performance. Models were optimized for binary crossentropy loss using the Adam optimizer^63^ with an initial learning rate of 0.0001. We reduced the learning rate by a factor of 0.5 when the validation loss plateaued for more than two epochs. We also stopped training when the validation loss failed to improve by more than 0.001 for twenty consecutive epochs. These two adjustments were made using the ReduceLROnPlateau and EarlyStopping callbacks in Tensorflow 2^61^ respectively. The training and validation loss curves for both the CNN-i and CNN-i + q models are depicted in Fig. 6. While the CNN-i + q model achieved better validation loss, it did not outperform the CNN-i model on the held out gold standard dataset.

**Fig. 7:**
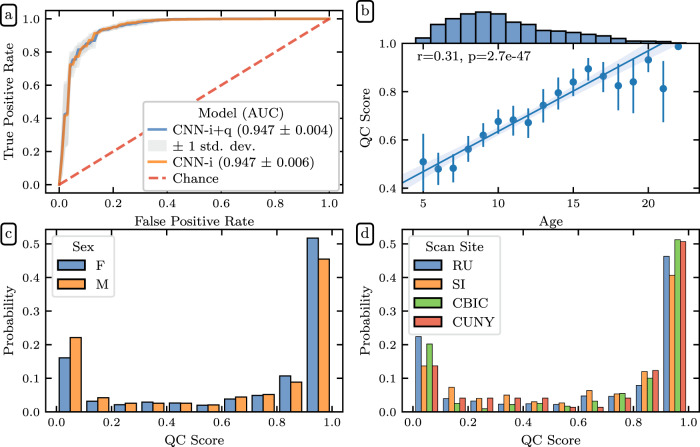
Deep learning QC scores: (**a**) ROC curves for two deep learning models trained on imaging data: one trained with additional automated data quality metrics from *QSIPrep* (blue) and one trained without (orange). The models performed roughly identically, reflecting that the data quality metrics are derived from the imaging data and are therefore redundant. Both outperformed the XGB-q predictions, indicating the added value of the diffusion weighted images. However, both models underperformed the XGB predictions, which also incorporate information from *Fibr* ratings for each scan. The error bands represent one standard deviation from the mean of the cross-validation splits. **(b)** Joint distributions showing a strong direct association between age and QC score (Pearson CC: 0.31). This likely reflects the well-known negative association between age and head motion in pediatric neuroimaging. The dots encode the mean QC score for each year of age with error bands representing a bootstrapped 95% confidence interval. The line depicts a linear regression relating age and QC score with translucent bands encoding a bootstrapped 95% confidence interval. Histograms showing the relationship between participants QC scores and their sex **(c)** and scan site **(d)**. QC distributions are independent of sex and scanning site.

The two models performed nearly identically and achieved an ROC-AUC of 0.947 ± 0.004 (Fig. 7a). The near-identical performance suggests that *QSIPrep*’s automated data quality metrics provided information that was redundant with information available in the imaging data. Both CNN-i and CNN-i + q outperformed XGB-q, which was trained only on automated QC metrics, but both modestly underperformed relative to the full XGB model, that uses *Fibr* scores in addition to the *QSIPrep* data quality metrics.

The openly available HBN-POD2 data released with this paper provides four QC ratings: the mean expert QC ratings, XGB-q and XGB predicted scores, as well as the CNN-i predicted score. However, we treat the CNN-i score as the definitive QC score because it is available for all participants, can be easily calculated for new participants in future HBN releases, and is more accurate than XGB-q in predicting expert ratings in the “gold standard” report set. When we refer to a participant’s QC score without specifying a generating model, the CNN-i score is assumed. Figure 7 depicts the distribution of these QC scores by age (Fig. 7b), sex (Fig. 7c), and scanning site (Fig. 7d). QC distributions are similar for each scan site and for male and female participants. Responses for the sex variable in HBN phenotypic data are limited to “male” and “female.”

#### Tractometry

**Fig. 8:**
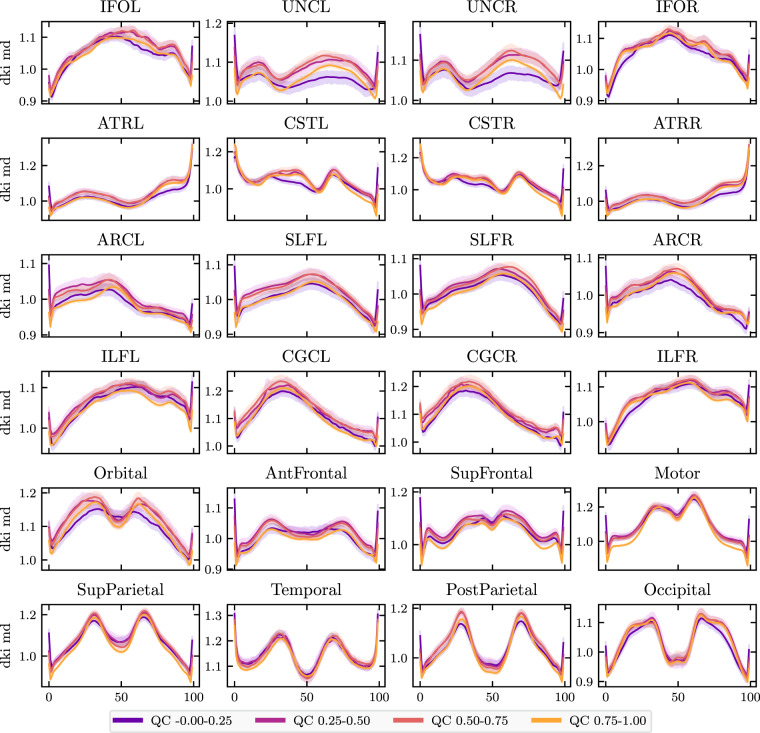
MD bundle profiles show large QC group differences: MD profiles binned by QC score in twenty-four major while matter bundles. The *x*-axis represents distance along the length of the fiber bundle. The left and right uncinate bundles were the most sensitive to QC score. Generally, QC score tended to flatten bundle profiles. Error bands represent bootstrapped 95% confidence intervals. Bundle abbreviations for lateralized bundles contain a trailing “L” or “R” indicating the hemisphere. Bundle abbreviations: inferior fronto-occipital fasciculus (IFO), uncinate (UNC), anterior thalamic radiation (ATR), corticospinal tract (CST), arcuate fasciculus (ARC), superior longitudinal fasciculus (SLF). inferior longitudinal fasciculus (ILF), cingulum cingulate (CGC), orbital corpus callosum (Orbital), anterior frontal corpus callosum (AntFrontal), superior frontal corpus callosum (SupFrontal), motor corpus callosum (Motor), superior parietal corpus callosum (SupParietal), temporal corpus callosum (Temporal), posterior parietal corpus callosum (PostParietal), and occipital corpus callosum (Occipital).

To further validate the importance of quality control, we used tract profiling^64–68^, which is a subset of tractometry^65,69^. In particular, tract profiling uses the results of dMRI tractography to quantify properties of the white matter along major pathways. We used the Python Automated Fiber Quantification toolbox (pyAFQ) as previously described^68^. Briefly, probabilistic tractography was performed using constrained spherical deconvolution fiber orientation distribution functions^70^, as implemented in DIPY^45^. Twenty-four major tracts, which are enumerated in Fig. 8, were identified using multiple criteria: inclusion ROIs and exclusion ROIs^71^, combined with a probabilistic atlas^72^. Each streamline was resampled to 100 nodes and the robust mean at each location was calculated by estimating the 3D covariance of the location of each node and excluding streamlines that are more than 5 standard deviations from the mean location in any node. Finally, a bundle profile of tissue properties in each bundle was created by interpolating the value of MRI maps of these tissue properties to the location of the nodes of the resampled streamlines designated to each bundle. In each of 100 nodes, the values were summed across streamlines, weighting the contribution of each streamline by the inverse of the Mahalanobis distance of the node from the average of that node across streamlines. Bundle profiles of mean diffusivity (MD) and fractional anisotropy (FA) from the diffusional kurtosis imaging (DKI) model^73^, implemented in DIPY^74^, were used in technical validation of the data and evaluation of the impacts of QC. We used the previously mentioned *cloudknot* cloud-computing library^46^ to parallelize the pyAFQ tractometry pipeline over individual participants on spot instances in the Amazon Web Services Batch service.

**Fig. 9:**
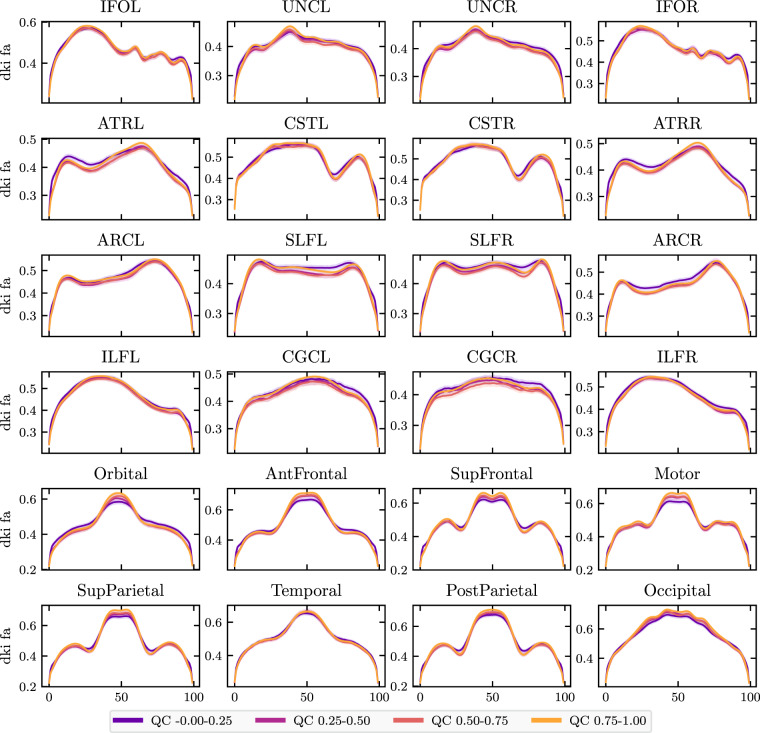
FA bundle profiles binned by QC score: FA profiles binned by QC score in twenty-four major while matter bundles. The *x*-axis represents distance along the length of the fiber bundle. Error bands represent bootstrapped 95% confidence intervals. Bundle abbreviations are as in Fig. 8.

Here, we plot mean diffusivity tract profiles (MD, Fig. 8) and fractional anisotropy profiles (FA, Fig. 9) grouped into four QC bins along the length of twenty-four bundles. While some bundles, such as the cingulum cingulate (CGC) and the inferior longitudinal fasciculus (ILF), appear insensitive to QC score, others, such as the uncinate (UNC) and the orbital portion of the corpus callosum, exhibit strong differences between QC bins. In most bundles, low QC scores tend to flatten the MD profile, indicating that MD appears artifactually homogeneous across the bundle.

## Data Records

### Curated imaging data

**Fig. 10:**
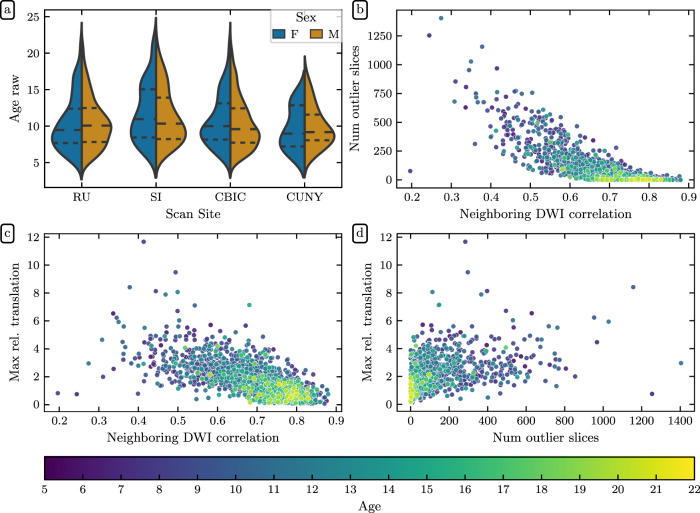
Demographic and *QSIPrep* quality metric distributions: (**a**) HBN age distributions by sex for each scanning site. Dashed lines indicate age quartiles. The remaining plots show associations between **(b)** neighboring diffusion-weighted imaging (DWI) correlation^17^ and the number of outlier slices, **(c)** neighboring DWI correlation and maximum relative translation, and **(d)** the number of outlier slices and maximum relative translation. The number of outlier slices is positively associated with the maximum relative translation, while neighboring DWI correlation is negatively associated with the other two metrics. These plots are colored by age, and reveal that older participants generally have higher quality data.

Curated BIDS data and their corresponding *QSIPrep* outputs are public resources that can be accessed by anyone using DataLad^31^ or standard Amazon Simple Storage Service (S3) access tools. The curated data are available in the FCP-INDI S3 bucket and as a DataLad dataset^9^ as indicated in Table 3. Likewise, the *QSIPrep* derivatives are available on FCP-INDI, as a standalone DataLad dataset^75^, and as a derivative subdataset in the primary HBN-POD2 DataLad dataset^9^. These processed diffusion derivatives are standard *QSIPrep* outputs (see https://qsiprep.readthedocs.io/en/latest/preprocessing.html#outputs-of-qsiprep), which contain preprocessed imaging data along with the corresponding QC metrics:

- *Anatomical Data* Preprocessed images, segmentations and transforms for spatial normalization are located in the anat/ directory of each session. The gray matter, white matter and cerebrospinal fluid (GM,WM, CSF) probabilistic segmentations are provided in nifti format with the _probtissue suffix. The deterministic segmentation is in _dseg.nii.gz. All images are in alignment with AC-PC-aligned sub-X_desc-preproc_T1w.nii.gz image unless they have space-MNI152NLin2009cAsym in their file name, in which case they are aligned to the MNI Nonlinear T1-weighted asymmetric brain template (version 2009c)^36^. The spatial transform between the AC-PC T1w image and MNI space is in the ITK/ANTs format file named sub-X_from-MNI152NLin2009cAsym_to-T1w_mode-image_xfm.h5. The brain mask from ANTsBrainExtraction.sh is included in the file with the _desc-brain_mask.nii.gz suffix.
- *Diffusion* *Data*. The preprocessed dMRI scan and accompanying metadata are in the dwi directory of each session. The fully-preprocessed dMRI data is follows the naming pattern sub-X_space-T1w_desc-preproc_dwi.nii.gz. These images all have an isotropic voxel size of 1.7 mm^3^ and are aligned in world coordinates with the anatomical image located at anat/sub-X_desc-preproc_T1w.nii.gz. Gradient information is provided in bval/bvec format compatible with DIPY and DSI Studio and the .b format compatible with MRtrix3. Volume-wise QC metrics including head motion parameters are included in the confounds.tsv file. Automatically computed quality measures for the entire image series are provided in the ImageQC.csv file, which includes the neighboring DWI Correlation, number of bad slices and head motion summary statistics. Figure 10 depicts pairwise distributions for the three of these automated data quality metrics that were most informative in QC models described later (see Tables 1 and 2). The desc-brain_mask file is a dMRI-based brain mask that should only be used when the T1w-based brain mask is inappropriate (i.e., when no susceptibility distortion correction has been applied).

**Table 3:** HBN-POD2 data records.

Data Resource	Repositories	Location
BIDS Curated Imaging	FCP-INDI^†^	/
	DataLad dataset^◊^	/
*QSIPrep* preprocessed DWI	FCP-INDI^†^	/derivatives/qsiprep/
	DataLad dataset^◊^	/derivatives/qsiprep/
	*QSIPrep* dataset^⨝^	/
CuBIDS variants	participants*	site_variant column
Raw expert ratings	OSF^‡^	/expert-qc/
Expert QC scores	participants*	expert_qc_score column
Raw community ratings	OSF^‡^	/community-qc/
Community QC scores	participants*	xgb_qc_scorecolumn
QSIQC QC scores	participants*	xgb_qsiprep_qc_score column
QSIQC model	GitHub	10.5281/zenodo.5949269
Deep learning input images	FCP-INDI^†^	/derivatives/qsiprep/derivatives/dlqc/
Deep learning models	OSF^‡^	/deep-learning-qc/saved-models
Deep learning QC scores	participants*	dl_qc_score column
Deep learning attributions	OSF^‡^	/deep-learning-qc/integrated-gradients
AFQ tractography & tractometry	FCP-INDI^†^	/derivatives/afq/
	DataLad dataset^◊^	/derivatives/afq/
	*AFQ* dataset^□^	/
AFQ streamline counts	FCP-INDI^†^	/derivatives/afq/participants.tsv
	DataLad dataset^◊^	/derivatives/afq/participants.tsv
	*AFQ* dataset^□^	/participants.tsv
AFQ tract profiles	FCP-INDI^†^	/derivatives/afq/combined_tract_profiles.csv
	DataLad dataset^◊^	/derivatives/afq/combined_tract_profiles.csv
	*AFQ* dataset^□^	/combined_tract_profiles.csv

^†^FCP-INDI:

All paths are relative to the root s3://fcp-indi/data/Projects/HBN/BIDS_curated/.

E.g., use the AWS CLI: aws s3 ls s3://fcp-indi/data/Projects/HBN/BIDS_curated/, or view these files in a web browser at

https://fcp-indi.s3.amazonaws.com/index.html#data/Projects/HBN/BIDS_curated/

^◊^HBN-POD2 DataLad dataset^9^:

Use datalad clone git@github.com:nrdg/HBN-POD2.git.

All paths are relative to the repository root.

^⨝^*QSIPrep* derivatives dataset^75^:

Use datalad clone git@github.com:nrdg/HBN-POD2-derivatives-qsiprep.git.

All paths are relative to the repository root.

^□^*AFQ* derivatives dataset^76^:

use datalad clone git@github.com:nrdg/HBN-POD2-derivatives-afq.git.

All paths are relative to the repository root.

^*^Participants.tsv: located on FCP-INDI and in the HBN-POD2 DataLad dataset at relative path derivatives/qsiprep/participants.tsv, and in the HBN-POD2 *QSIPrep* derivatives DataLad

dataset at participants.tsv

^‡^HBN-POD2 OSF Project^118^: all paths are relative to the root HBN-POD2 QC/OSF Storage.

### CuBIDS Variants

We identified 20 unique dMRI acquisitions across HBN-POD2, which are summarized in Table 4. Site CBIC has two acquisition types: “64dir,” which shares its pulse sequence with sites RU and CUNY, and “ABCD64dir,” with acquisition parameters that better match the ABCD study (TE = 0.089 s and TR = 4.1 s). The “Most_Common” variant identifies the most common combination of acquisition parameters for a given site and acquisition. The “Low_Volume” variant identifies participants from all sites with less than 129 DWI volumes, which is the number of volumes in the most common variants. All remaining variant names identify the acquisition parameter(s) that differ from those of the most common variant. For example, the “MultibandAccelerationFactor” variant has a different multiband acceleration factor than that of the the most common variant but all participants within that variant share the same multiband acceleration factor. Variants that differ by multiple acquisition parameters have names that are composed of concatenated parameters. For example, the variant “Dim3SizeVoxelSizeDim3” varies both in the number of voxels in dimension 3 (“Dim3Size”) and in the voxel size in dimension 3 (“VoxelSizeDim3”).

**Table 4:** Participant counts for HBN-POD2 variants.

Site	Acquisition	Variant	Count
CBIC	64dir	Most_Common	828
CBIC	64dir	Obliquity	32
CBIC	64dir	VoxelSizeDim1VoxelSizeDim2	1
CBIC	ABCD64dir	Most_Common	15
CBIC	ABCD64dir	HasFmap	2
CBIC	ABCD64dir	MultibandAccelerationFactor	1
CBIC	ABCD64dir	Obliquity	1
CUNY	64dir	Most_Common	68
CUNY	64dir	Dim3SizeVoxelSizeDim3	4
CUNY	64dir	Obliquity	2
RU	64dir	Most_Common	859
RU	64dir	NoFmap	5
RU	64dir	Obliquity	8
RU	64dir	PhaseEncodingDirection	1
SI	64dir	EchoTime	1
SI	64dir	EchoTimePhaseEncodingDirection	9
SI	64dir	Most_Common	269
SI	64dir	NoFmap	2
SI	64dir	Obliquity	12
All Sites	All Acquisitions	Low_Volume_Count	14

The specific variant of each scanning session is provided as a column in the HBN-POD2 participant.tsv file. Users may use this information to test their BIDS-Apps on a subset of participants that represent the full range of acquisition parameters that are present.

### Quality control data

We provide four separate QC scores in the participants.tsv file described in Table 3. The mean expert ratings are available in the “expert_qc_score” column. These ratings are scaled to the range 0 to 1, so that a mean rating from 0 to 0.2 corresponds to an expert rating of “definitely fail”, a mean rating from 0.2 to 0.4 corresponds to “probably fail”, from 0.4 to 0.6 corresponds to “not sure”, from 0.6 to 0.8 corresponds to “probably pass”, and 0.8 to 1.0 corresponds to “definitely pass.” The XGB model’s positive class probabilities are available in the “xgb_qc_score” column, while the XGB-q model’s positive class probabilities are available in the “xgb_qsiprep_qc_score” column. Finally, the CNN-i + q model’s positive class probabilities are available in the “dl_qc_score” column.

### Tractography and tractometry

The outputs of the pyAFQ tractometry pipeline, including tractography and tract profiles, are provided as specified in Table reftab:data-records: in a BIDS derivative directory in the FCP-INDI AWS S3 bucket, as a Datalad dataset^76^ and as a DataLad subdataset in the primary HBN-POD2 dataset^9^. In particular the FA and MD tract profiles for each participants are available on S3 at s3://fcp-indi/data/Projects/HBN/BIDS_curated/derivatives/afq/combined_tract_profiles.csv. Streamline counts for each of the bundles are available at s3://fcp-indi/data/Projects/HBN/BIDS_curated/derivatives/afq/participants.tsv.

For each subject, intermediate data derivatives of the pyAFQ pipeline are also provided.

- A brain mask and mean *b* = 0 image are saved with “_brain_mask.nii.gz” and “_b0.nii.gz” file-name suffixes. A set of diffusion modeling derivatives are saved for each of three different diffusion models: DTI, DKI and CSD. Diffusion model parameters are saved with the “_diffmodel.nii.gz” suffix. Derived model scalars are saved with suffixes that indicate the model and the scalar. For example, the FA derived from the DTI model is saved with the “_DTI_FA.nii.gz” suffix.
- Masks used to initialize tractography are saved with the “seed_mask.nii.gz” suffix, while those used to determine the stopping criterion for tractography are stored with the “stop_mask.nii.gz” suffix.
- Files that define a non-linear transformation between the individual subject anatomy and the MNI template for the purpose of waypoint ROI placement are stored with “mapping_from-DWI_to_MNI_xfm.nii.gz” (non-linear component) and “prealing_from-DWI_to_MNI_xfm.npy” (affine component) suffixes. The waypoint ROIs, transformed to the subject anatomy through this non-linear transformation are also stored in the “ROIs” sub-directory.
- Tractography derivatives are stored with the “_tractography.trk”. The whole-brain tractography, which serves as the input data for bundle segmentation, is stored with the “_CSD_desc-prob_tractography.trk” suffix. Streamlines that were selected for inclusion in one of the major bundles are stored in separate files in the “bundles” sub-directory and saved in a consolidated file with the “CSD_desc-prob-afq_tractography.trk” suffix. The streamlines selected for inclusion and also additionally cleaned through a process of outlier removal are stored with the “CSD_desc-prob-afq-clean_tractography.trk” suffix and also in a “clean_bundles” sub-directory.
- An interactive visualization of bundles relative to the individual anatomy is stored with the “_viz.html” suffix and summaries of streamline counts in each bundle are stored with the “_sl_count.csv”. Additional visualizations are provided in the “tract_profile_plots” and “viz_bundles” sub-directory.
- Individual tract profiles are stored with the “afq_profiles.csv” suffix. This information is redundant with the one provided in aggregate format in the “combined_tract_profiles.csv” file.
- Individual streamline counts for each of the bundles are stored with the “_sl_count.csv” suffix. This information is redundant with the one provided in aggregate format in the “participants.tsv” file.

## Technical Validation

### Attribution masks for the deep learning classifier

We generated post-hoc attribution maps that highlight regions of the input volume that are relevant for the deep learning generated QC scores. The integrated gradient method^26^ is a gradient-based attribution method^77^ that aggregates gradients for synthetic images interpolating between a baseline image and the input image. It has been used to interpret deep learning models applied to retinal imaging in diabetic retinopathy^78^ and glaucoma^79^ prediction, as well as in multiple sclerosis prediction from brain MRI^80^. Our goal is to confirm that the CNN-i model was driven by the same features that would drive the expert rating, thereby bolstering the decision to apply it to new data.

To generate the attribution maps, we followed Tensorflow’s integrated gradients tutorial^81^ with a black baseline image and 128 steps in the Riemann sum approximation of the integral (i.e., m_steps = 128).

**Fig. 11:**
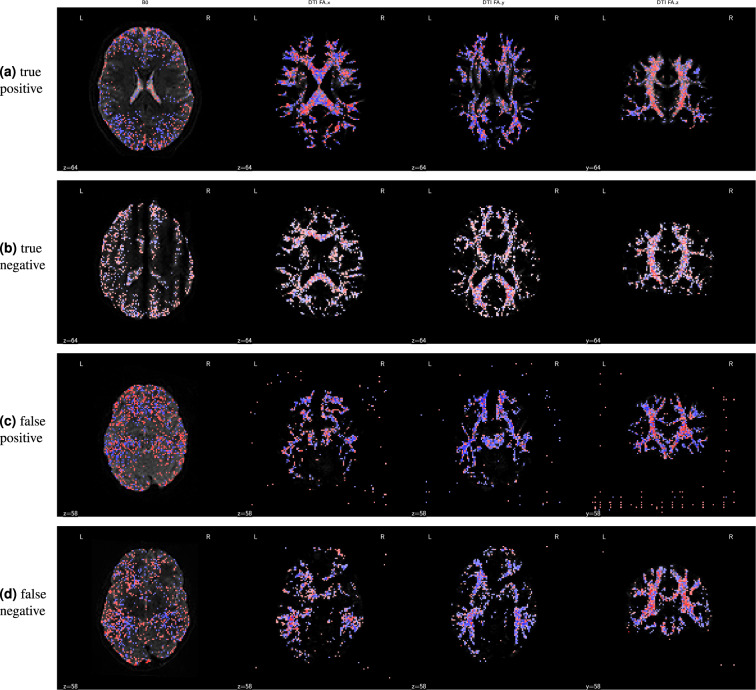
Integrated gradient attribution maps for the deep learning classifier: Each column depicts a different channel of the input tensor: the *b* = 0 DWI volume and the DEC-FA images in the *x*, *y*, and *z* directions. The first three columns show an axial slice while the last column shows a coronal slice. Blue voxels indicate positive attribution (i.e., evidence for passing the participant), while red voxels indicate negative attribution (i.e., evidence for QC failure). The voxels with small magnitude attribution values (≤98% of the highest value in each image) have been rendered to be transparent, as they do not indicate strong evidence in either direction. In these cases, the underlying grayscale depicts the input channel (*b* = 0 or *x*, *y*, or *z* elements of the DEC-FA image). Each row depicts a representative participant from each confusion class: **(a)** Attribution maps for a true positive prediction. The model looked at the entire brain and focused on known white matter bundles in the DEC-FA channels. In particular, it focused on lateral bundles in the *x* direction, anterior-posterior bundles in the *y* direction, and superior-inferior bundles in the *z* direction. **(b)** Attribution maps for a true negative prediction. The model focused primarily on the *b* = 0 channel, suggesting that it ignores DEC-FA when motion artifacts like banding are present. **(c)** Attribution maps for a false positive prediction. Both the false positive and negative predictions were low confidence predictions. This is reinforced by the fact that the model viewed some voxels that are outside of the brain as just as informative as those in major white matter tracts. **(d)** Attribution maps for a false negative prediction. The model failed to find long-range white matter tracts in the anterior-posterior and lateral directions. We also speculate that the model expected left-right symmetry in the DEC-FA channels and assigned negative attribution to asymmetrical features.

Figure 11 shows attribution maps for example participants from each confusion class: true positive, true negative, false positive, and false negative. The columns correspond to the different channels of the deep learning input volume: the *b* = 0 reference image and the DEC-FA in the *x*, *y*, and *z* directions. These integrated gradients are dimensionless quantities but their sign is meaningful. They are proportional to the probability of assigning one label (“pass”) or another (“fail”). The blue voxels indicate positive attribution, that is, data that supports a passing QC classification. Conversely, the red voxels indicate negative attribution, data that supports a failing QC classification. The true positive map indicates that the network was looking at the entire brain rather than focusing on any one anatomical region (Fig. 11a). Moreover, the model identified white matter fascicles that travel along the direction of the input channel: lateral for *x*, anterior-posterior for *y*, and superior-inferior for *z*. The true negative attribution map (Fig. 11b) reveals that when the reference *b* = 0 volume contains motion artifacts, such as banding, the network ignored the otherwise positive attributions for the clearly identifiable white matter tracts in the DEC-FA channels. The false positive map (Fig. 11c) and the false negative map (Fig. 11d) should be interpreted differently since they come from low confidence predictions; the probability of passing hovered on either side of the pass/fail threshold. For example, in the false positive case, the network was confused enough that it treated voxels that are outside of the brain to be as informative as voxels in the major white matter bundles.

### QC prediction models can generalize to unseen sites

**Fig. 12:**
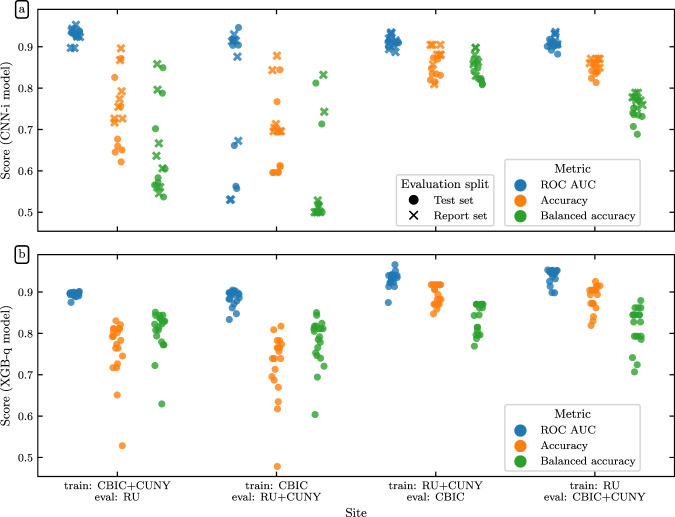
Generalization of QC scores to unseen sites: In each experiment, CNN-i (**a**) and XGB-q (**b**) models were trained with some sites held out and evaluated only on data from these held out sites. Model performance is quantified as ROC-AUC (blue), accuracy (orange) and balanced accuracy (green). For XGB-q, the targets are the expert ratings on data from the held out site. For CNN-i, performance is scored against XGB scores (as used before; test set in filled circles), or expert ratings on the data from the held out site (report set in crosses). Summary statistics for this plot are listed in Table 5.

Site harmonization is a major issue for any multisite neuroimaging study and developing automated QC tools that generalize between sites has been a perennial issue^82^. Furthermore, the ability to generalize between sites in a single multisite study would signal the promise of generalizing to other datasets altogether. To better understand the ability of our QC models to generalize across scanning sites, we trained multiple versions of XGB-q and CNN-i on partitions of the data with different scanning sites held out and then evaluated those models on the held out sites (Fig. 12 and Table 5). These models were therefore evaluated on data from “unseen” sites. We constructed these train/evaluate splits from combinations of the HBN sites with 3 T scanners (RU, CBIC, and CUNY), and excluded CUNY as a standalone training or test site because of its low number of participants (*N* = 74). This left four combinations of site-generated training splits: CBIC + CUNY (eval: RU), CBIC (eval: RU + CUNY), RU + CUNY (eval: CBIC), and RU (eval: CBIC + CUNY).

**Table 5:** Site generalization summary statistics: Below we list the mean ± standard deviation of the site generalization evaluation metrics displayed in Fig. 12.

Model	Site	Accuracy	Balanced accuracy	ROC-AUC
CNN-i	train: CBIC + CUNY, test: RU	0.748 ± 0.086	0.652 ± 0.112	0.930 ± 0.015
	train: CBIC, test: RU + CUNY	0.696 ± 0.095	0.574 ± 0.123	0.791 ± 0.169
	train: RU + CUNY, test: CBIC	0.859 ± 0.033	0.847 ± 0.030	0.912 ± 0.013
	train: RU, test: CBIC + CUNY	0.851 ± 0.018	0.753 ± 0.029	0.910 ± 0.014
XGB-q	train: CBIC + CUNY, test: RU	0.763 ± 0.071	0.805 ± 0.052	0.895 ± 0.006
	train: CBIC, test: RU + CUNY	0.725 ± 0.079	0.779 ± 0.058	0.886 ± 0.019
	train: RU + CUNY, test: CBIC	0.894 ± 0.024	0.838 ± 0.036	0.931 ± 0.018
	train: RU, test: CBIC + CUNY	0.886 ± 0.030	0.816 ± 0.048	0.940 ± 0.017

For each of the CNN-i and XGB-q model families and each of the site generalization splits, we report the accuracy, balanced accuracy, and ROC-AUC.

We trained eight models (with distinct random seeds) from the CNN-i family of models using the global XGB scores as targets, just as with the full CNN-i model. Similarly, we trained twenty models (with distinct random seeds) from the XGB-q family of models using the expert scores as targets, just as with the full XGB-q model. For each model, we reported three evaluation metrics: ROC-AUC, accuracy, and balanced accuracy. Because the distribution of QC scores was imbalanced (Figs. 2a and 7d), we included balanced accuracy as an evaluation metric. Balanced accuracy avoids inflated accuracy estimates on imbalanced data^83^, and in the binary classification case, it is the mean of the sensitivity and specificity. For the CNN-i family, we further decomposed the evaluation split into a report set, for which expert scores were available, and a test set, with participants who were not in the “gold standard” dataset. For the report set, we evaluated the model using the expert scores as the ground truth. For the test set, we evaluated each model using the XGB scores as ground truth. Aside from the specification of train and evaluation splits, model training followed exactly the same procedure as for the full dataset. For example, we use the same cross validation and hyperparameter optimization procedure for the XGB-q family as for the original XGB-q model and the same architecture, input format, and early stopping criteria for the CNN-i family as for the CNN-i model.

ROC-AUC for generalization is uniformly high for both the XGB-q and the CNN-i models. However, more importantly, accuracy and balanced accuracy vary substantially: depending on the site that was used for training, balanced accuracy could be as low as guess rate, particularly for the CNN-i model. Notably, it seems that including the RU site in the training data led to relatively high balanced accuracy in both models. The XGB-q model balanced accuracy was less dependent on the specific sites used for training, but also displayed some variability across permutations of this experiment. In particular, the benefit from including the “right site” in the training data, namely RU, eclipsed the slight benefit conferred by including more than one site in the training data.

### Quality control improves inference

To demonstrate the effect that quality control has on inference, we analyzed tract profile data derived from HBN-POD2 data.

Missing values were imputed using median imputation as implemented by *scikit-learn*’s SimpleImputer class. Because the HBN-POD2 bundle profiles exhibit strong site effects^84^, we used the ComBat harmonization method to robustly adjust for site effects in the tract profiles^85–88^, using the *neurocombat_sklearn* library^89^.

**Fig. 13:**
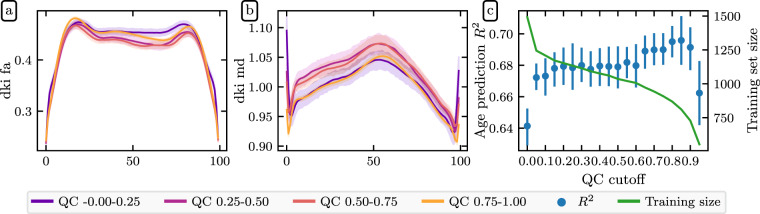
Imposing a QC cutoff improves age prediction: Cross validated *R*^2^ scores (left axis, blue dots) from an age prediction model increase after screening participants by QC score. We see the most dramatic increase in *R*^2^ after imposing even the lowest cutoff of 0.05. Thereafter, the *R*^2^ scores trend upward until a cutoff of ~0.95, where the training set size (right axis, orange line) becomes too small to sustain model performance. The error bands represent a bootstrapped 95% confidence interval.

In Fig. 13, we plot the mean diffusivity (MD) and fractional anisotropy (FA) profiles along the left superior longitudinal fasciculus (SLFL) grouped into four QC bins. The SLFL exhibits strong differences between QC bins. Low QC scores tend to flatten the MD and FA profiles, indicating that MD and FA appear artifactually homogeneous across the bundle.

The effect of QC score on white matter bundle profiles indicates that researchers using HBN-POD2 should incorporate QC in their analyses, either by applying a QC cutoff when selecting participants or by explicitly adding QC score to their inferential models. Failure to do so may cause spurious associations or degrade predictive performance. To demonstrate this, we selected participant age as a representative phenotypic benchmark because (i) it operates on a natural scale with meaningful units and (ii) despite the unique methodological challenges it presents for biomarker identification^90^, brain age prediction may be diagnostic of overall brain health^84,91,92^. We observed the effect of varying QC cutoff on the predictive performance of an age prediction model (Fig. 13).

We evaluated this effect by observing cross-validated *R*^2^ values of gradient boosted trees models implemented using XGBoost. The input feature space for each model consisted of 4,800 features per participant, comprising 100 nodes for each of MD and FA in the twenty-four major tracts. We imputed missing bundles and harmonized the different scanning sites as above. The XGBoost models’ hyperparameters were hand-tuned to values that have been performant in the authors’ previous experience. Within the limited age range of the HBN study, MD and FA follow logarithmic maturation trajectories^93^. We therefore log-transformed each participant’s age before prediction using the TransformedTargetRegressor class from *scikit-learn*. For each value of the QC cutoff between 0 and 0.95, in steps of 0.05, we computed the cross-validated *R*^2^ values using *scikit-learn*’s cross_val_score function with repeated K-fold cross-validation using five folds and five repeats.

Cross-validated *R*^2^ scores for an age prediction model varied depending on the QC cutoff (Fig. 13). An initial large improvement was achieved by excluding the 200 participants with the lowest QC scores, followed by a gradual increase in performance. Finally, when a large number of participants is excluded, performance deteriorated again.

## Usage Notes

HBN-POD2 is one of the largest child and adolescent diffusion imaging datasets with preprocessed derivatives that is currently openly available. The dataset was designed to comply with the best practices of the field. For example, it complies with the current draft of the BIDS diffusion derivative specification^94^. It will grow continuously as the HBN study acquires more data, eventually reaching its 5,000 participant goal.

### Preprocessing and quality control increase the impact of openly-available data

The HBN-POD2 data is amenable to many different analyses, including tractometry^64,68,95^, graph theoretical analysis^96^, and combinations with functional MRI data and other data types for the same participants. The availability of standardized preprocessed diffusion data will allow researchers to create and test hypotheses on the white matter properties underlying behavior and disease, from reading and math acquisition to childhood adversity and mental health. As such, this dataset will accelerate discovery at the nexus of white matter microstructure and neurodevelopmental and learning disorders.

In large developmental datasets, it is critically important to perform accurate and reliable QC of the data. QC is associated not just with age, but with many phenotypic variables of interest in cognition and psychopathology^97^. HBN-POD2 provides four separate QC scores alongside its large dataset of pediatric neuroimaging diffusion derivatives, paving the way for users of the data to incorporate considerations of data quality into their analysis of the processed data. Unsurprisingly, QC scores are strongly correlated with age (Fig. 7). This accords with the negative association between head motion and age in developmental studies, which is well established both in general^98–101^ and specifically for resting-state fMRI in the HBN dataset^5,6^. Moreover, it is important that QC has bundle-specific and spatially localized effects (Fig. 8). Analysis of this data that does not incorporate QC is likely to find replicable but invalid effects. For example, in patient-control studies, patients are likely to have lower quality data. And analysis of such patient data that does not control for QC will find spatially-localized and replicable group differences that are due to data quality, not necessarily underlying neuroanatomical differences.

We further demonstrated the impact of QC in a benchmark age prediction task (Fig. 13). In this case, the increase in model performance from imposing a QC cutoff is intuitive: we know from Fig. 8 that participants with low QC scores have reduced MD, but MD also decreases as participants mature^84,93^. Eliminating participants with low QC therefore removes the ones who may look artificially older from the analysis, improving overall performance. The most noticeable improvement in performance comes after imposing the most modest cutoff of 0.05, suggesting that inferences may benefit from *any* QC screening. On the other hand, QC screening inherently introduces a tradeoff between the desire for high quality data and the desire for a large sample size. In this case, after a QC cutoff of around 0.9, the training set size is reduced such that it degrades predictive performance. Importantly, we do not expect the sensitivity analysis of an age prediction model to generalize to other analyses and therefore recommend that researchers using HBN-POD2 choose the most appropriate QC cutoff for their research question and consider including QC score as a model covariate in their analyses.

### Automated quality control: scalability, interpretability, and generalization

The predictive performance of the CNN-i model (Fig. 7a) gives us confidence that it could accurately classify unseen data from the same sites, justifying its extension to the entire HBN-POD2 dataset and to future releases of HBN. However, one limitation of this model is that it does not satisfactorily explain its decisions. As deep learning models have been increasingly applied to medical image analysis, there is an evolving interest in the interpretability of these models^23–25,102^. While an exhaustive interpretation of deep learning QC models is beyond the scope of this work, we provided a preliminary qualitative interpretation of the CNN-i model (Fig. 11) that demonstrates the intuitive nature of its decisions.

The accuracy in generalizing to unseen data from HBN also suggested the tantalizing possibility that the QC models would be able to generalize to similar data from other datasets. To assess this, we trained the models with unseen sites held out (Fig. 12). Both the CNN-i model and the XGB-q model do sometimes generalize to data from unseen sites, suggesting that they would be able to generalize to some other datasets as well. However, they do not reliably generalize, implying that they should not currently be used in this way. Future work could build upon the work that we have done here to establish a procedure whereby the models that we fit in HBN would be applied to data from other studies, but comprehensive calibration and validation would have to be undertaken as part of this procedure.

We recognize that decisions about QC exclusion/inclusion must balance accuracy, interpretability, generalization to new data, and scalability to ever larger datasets. We therefore provide three additional scores: (i) the mean expert QC score for the 200 participants in the gold standard dataset, (ii) the scores predicted by the XGB model, which outperformed all other models when evaluated against the gold standard ratings, but which are only available for participants that have community science scores, and (iii) the scores predicted by the XGB-q model, which underperformed the deep learning generated scores, but which rely only on the automated QC metrics output by *QSIPrep*. We view the XGB-q scores, which are available for all participants, as a more interpretable and scalable fallback because the XGB-q model ingests *QSIPrep* output without any further postprocessing. XGB-q also provides slightly more uniform performance in generalization to unseen HBN sites (Fig. 12). Because the XGB-q model most readily generalizes to other *QSIPrep* outputs, we packaged it as an independent QC service in the QSIQC software package^103^, available both as a docker image at ghcr.io/richford/qsiqc and as a Streamlit app at https://share.streamlit.io/richford/qsiqc/main/app.py. The decision to use a more interpretable but slightly less performant method of generating QC scores was also advocated by Tobe *et al*.^104^, who noted that the Euler number of T1-weighed images^105^ in the NKI-Rockland dataset can reliably predict scores generated with *Braindr*, the community science application developed in our previous work^22^.

We also note that the issue of algorithmic impact in choosing a QC method is not exclusive to the deep learning model. We have chosen models that most reliably reproduce the gold standard ratings, but a reliable algorithm might still negatively influence researcher’s decisions. For example, excluding participants by QC score could spur them to exclude populations deserving of study, as when QC score is highly correlated with age or socio-economic status. We therefore caution researchers to examine interactions between the QC scores we provide and their phenotype of interest.

More generally, QC in the dataset that we have produced is fundamentally anchored to the decisions made by the expert observers. While Cohen’s *κ* between some pairs of experts can be as low as 0.52, IRR quantified across all of the experts with ICC3k is excellent. Nevertheless, it is possible that improvements to the final QC scores could be obtained through improvements to IRR, or by designing a more extensive expert QC protocol. The tradeoff between more extensive QC for each participant and more superficial QC on more participants was not explored in this study, but could also be the target for future research.

Finally, the QC scores in this dataset are single scalar representations of the quality of each participant’s diffusion weighted imaging. They should not be taken as a single measurement of suitability for inclusion. QC metrics are exclusion metrics, not inclusion metrics. In fact, we postulate that no single measurement is suitable as an inclusion criterion *by itself*. For example, some HBN participants have both neuroanatomical abnormalities and high quality diffusion data, as measured by high neighboring DWI correlation, low framewise displacement, and high QC scores. Therefore, one would need to include other sources of information when considering inclusion in a particular study. For example, we recommend that users consult the pyAFQ streamline counts (see Table 3) to assess suitability for inclusion in a study of normative brains.

### Transparent pipelines provide an extensible baseline for future methods

While the primary audience of HBN-POD2 is researchers in neurodevelopment who will use the dMRI derivatives in their studies, other researchers may use HBN-POD2 to develop new preprocessing algorithms or quality control methods. In this respect, HBN-POD2 follows Avesani *et al*.^106^, who recognized the diverse interests that different scientific communities have in reusing neuroimaging data and coined the term *data upcycling* to promote multiple-use data sharing for purposes secondary to those of the original project. Complementing the approach taken in Avesani *et al*.‘s work, which provided dMRI from a small number of participants preprocessed with many pipelines, HBN-POD2 contains many participants, all processed with a single state of the art pipeline, *QSIPrep*. For researchers developing new preprocessing algorithms, HBN-POD2 provides a large, openly available baseline to which they can compare their results.

Similarly, neuroimaging QC methods developers will benefit from a large benchmark dataset of expert, community science, and automated QC ratings, with which to test new methods. Importantly, the architecture and parameters of the deep learning network used for QC are also provided as part of this work, allowing application of this network to future releases of HBN data, and allowing other researchers to build upon our efforts. Indeed, in this work, we have extended our previous work on what we now call “hybrid QC”. This approach, which we originally applied to the first two releases of the HBN T1-weighted data^22^ (using the *Braindr* web app: https://braindr.us) was extended here in several respects. First, the *Braindr* study used a smaller dataset of approximately 700 participants, while we extended this approach to well over 2000 participants. Second, *Braindr* relied on approximately 80000 ratings from 261 users. Here, we received more than 500000 ratings from 374 community scientists. As our understanding of the role of community scientist contributions has evolved, we decided that we would include as collective co-authors community scientists who contributed more than 3000 ratings^53^. Third, *Braindr* used data from only a single site. Here, multi-site data was used. This opens up multiple possibilities for deeper exploration of between-site quality differences, and also for harmonization of QC across sites, as we have attempted here. Last, the most challenging extension of hybrid QC from *Braindr* to this study entailed developing an approach that would encompass multi-volume dMRI data. On the one hand, this meant that the task performed by the expert observers was more challenging, because it required examination of the full dMRI time-series for every scan. To wit, expert inter-rater reliability was considerably higher for the T1-weighted only data in^22^ than for the dMRI data used (Fig. 2e). On the other hand, it also meant that the 4D data had to be summarized into 2D data to be displayed in the *Fibr* web application. This was achieved by summarizing the entire time-series as a DEC-FA + *b* = 0 image and presenting community scientists with animated sections of these images that showed how the data extended over several horizontal slices. In addition, the extension to 4D data required developing new deep learning architectures for analysis of 4D images, including upstream contributions to *Nobrainer*, a community-developed software library for deep learning in neuroimaging data^107^. These extensions demonstrate that the hybrid QC approach generalizes very well to a variety of different circumstances. Future applications of this approach could generalize to functional MRI data, as well as other large datasets from other kinds of measurements and other research domains.

### Future work and open problems

While our work was based on HBN releases 1–9, theThe HBN study plans to acquire imaging data for over 5000 participants, necessitating future data releases. In particular, the 10th release of HBN data was already made available between completion of the work and the publication of this paper. Since this 10th release as well as future releases of HBN will also require future releases of HBN-POD2, a plan for these is essential. This is a general issue affecting multi-year neuroimaging projects for which derivative data is being released before study completion. The use of *QSIPrep*, *cloudknot* and the containerization of the QC score assignment process facilitate running the exact pipeline described in this paper on newly released participants. However, this approach is somewhat unsatisfactory because it fails to anticipate improvements in preprocessing methodology. That is, what should we do when *QSIPrep* is inevitably updated between HBN releases? Enforce standardization by using an outdated pipeline or use state-of-the-art preprocessing at the expense of standardized processing between releases? Because the use of *cloudknot* and AWS Spot Instances renders preprocessing fast and relatively inexpensive, we propose a third way: if improvements to the preprocessing pipeline are available with a new HBN release, we plan to execute the improved pipeline on the entire HBN dataset, while preserving the previous baseline release in an archived BIDS derivative dataset.

Undertaking the processing and QC effort to generate HBN-POD2 required construction and deployment of substantial informatics infrastructure, including tools for cloud computing, web applications for expert annotation and for community science rating and analysis software. All of these tools are provided openly, so that this approach can be generalized even more widely in other projects and in other scientific fields.

## Data Availability

To facilitate replicability, Jupyter notebooks^108^ and Dockerfiles^109^ necessary to reproduce the methods described herein are provided in the HBN-POD2 GitHub repository at https://github.com/richford/hbn-pod2-qc. The specific version of the repository used in this study is documented in^110^. Most of the code in this repository uses Pandas^111,112^, Numpy^113^, Matplotlib^114^, and Seaborn^115^. The make or make help commands will list the available commands and make build will build the requisite Docker images to analyze HBN-POD2 QC data. In order to separate data from analysis code^116^, we provide intermediate data necessary to analyze the QC results in an OSF^117^ project^118^, the contents of which can be downloaded using the make data command in the root of the HBN-POD2 GitHub repository. The NIFTI-1 files and TFRecord files provided as input to the CNN models may be separately downloaded using the make niftis and make tfrecs commands, respectively. The remaining make commands and Jupyter notebooks follow the major steps of the methods section: 1. The *cloudknot* preprocessing function used to execute *QSIPrep* workflows on curated data was a thin wrapper around *QSIPrep*’s command line interface and is provided in the “notebooks” directory of the HBN-POD2 GitHub repository in a Jupyter notebook with the suffix preprocess-remaining-hbn-curated.ipynb. 2. The expert rating analysis can be replicated using the make expert-qc command in the HBN-POD2 GitHub repository. 3. The *Fibr* community science web application is based on the SwipesForScience framework (swipesforscience.org), which generates a web application for community science given an open repository of images to be labelled and a configuration file. The source code for the *Fibr* web application is available at https://github.com/richford/fibr. 4. The images that the *Fibr* raters saw were generated using a *DIPY*^45^ TensorModel in a *cloudknot*-enabled Jupyter notebook that is available in the “notebooks” directory of the *Fibr* GitHub repository. *Fibr* saves each community rating to its Google Firebase backend, the contents of which have been archived to the HBN-POD2 OSF project as specified in Table 3. 5. The community ratings analysis can be replicated using the make community-qc command in the HBN-POD2 GitHub repository. Saved model checkpoints for each of the XGB models are available in the HBN-POD2 OSF project and are automatically downloaded with the make data command. 6. The input multichannel volumes for the CNN models were generated using *DIPY*^45^ and *cloudknot*^46^ and saved as NIfTI-1 files^119^. These NIfTI files were then converted to the Tensorflow TFRecord format using the *Nobrainer* deep learning framework^107^. The Jupyter notebooks used to create these NIfTI and TFRecord files are available in the “notebooks” directory of the HBN-POD2 GitHub repository, with suffixes save-b0-tensorfa-nifti.ipynb and save-tfrecs.ipynb, respectively. 7. We trained the CNN models using the Google Cloud AI Platform Training service; the HBN-POD2 GitHub repository contains Docker services to launch training (with make dl-train) and prediction (with make dl-predict) jobs on Google Cloud, if the user has provided the appropriate credentials in an environment file and placed the TFRecord files on Google Cloud Storage. Further details on how to organize these files and write an environment file are available in the HBN-POD2 GitHub repository’s README_GCP.md file. To generate the figures depicting the deep learning QC pipeline and results, use the make deep-learning-figures command. 8. We provide a Docker service to compute integrated gradient attribution maps on Google Cloud, which can be invoked using the make dl-integrated-gradients command. This step also requires the setup steps described in README_GCP.md. 9. We provide a Docker service to conduct the CNN-i site generalization experiments on Google Cloud, which can be invoked using the make dl-site-generalization command, which, again, requires the setup steps described in README_GCP.md. Similarly, theThe XGB-q site generalization experiments can be replicated locally using the make site-generalization command, which will also plot the results of the CNN-i experiments. 10. The tractometry pipeline was executed using pyAFQ and *cloudknot* in a Jupyter notebook provided in the “notebooks” directory of the HBN-POD2 GitHub repository with the with suffix afq-hbn-curated.ipynb. with suffix afq-hbn-curated.ipynb, provided in the HBN-POD2 GitHub repository in the “notebooks” directory. The pyAFQ documentation contains a more pedagogical example of using pyAFQ with cloudknot to analyze a large openly available dataset (https://yeatmanlab.github.io/pyAFQ/auto_examples/cloudknot_example.html). 11. The bundle profile and age prediction analyses can be replicated using the make bundle-profiles and make inference commands, respectively.
