# Publisher Correction: An analysis-ready and quality controlled resource for pediatric brain white-matter research

**DOI:** 10.1038/s41597-022-01770-z

**Published:** 2022-10-31

**Authors:** Adam Richie-Halford, Matthew Cieslak, Lei Ai, Sendy Caffarra, Sydney Covitz, Alexandre R. Franco, Iliana I. Karipidis, John Kruper, Michael Milham, Bárbara Avelar-Pereira, Ethan Roy, Valerie J. Sydnor, Jason D. Yeatman, The Fibr Community Science Consortium, Nicholas J. Abbott, John A. E. Anderson, B. Gagana, MaryLena Bleile, Peter S. Bloomfield, Vince Bottom, Josiane Bourque, Rory Boyle, Julia K. Brynildsen, Navona Calarco, Jaime J. Castrellon, Natasha Chaku, Bosi Chen, Sidhant Chopra, Emily B. J. Coffey, Nigel Colenbier, Daniel J. Cox, James Elliott Crippen, Jacob J. Crouse, Szabolcs David, Benjamin De Leener, Gwyneth Delap, Zhi-De Deng, Jules Roger Dugre, Anders Eklund, Kirsten Ellis, Arielle Ered, Harry Farmer, Joshua Faskowitz, Jody E. Finch, Guillaume Flandin, Matthew W. Flounders, Leon Fonville, Summer B. Frandsen, Dea Garic, Patricia Garrido-Vásquez, Gabriel Gonzalez-Escamilla, Shannon E. Grogans, Mareike Grotheer, David C. Gruskin, Guido I. Guberman, Edda Briana Haggerty, Younghee Hahn, Elizabeth H. Hall, Jamie L. Hanson, Yann Harel, Bruno Hebling Vieira, Meike D. Hettwer, Harriet Hobday, Corey Horien, Fan Huang, Zeeshan M. Huque, Anthony R. James, Isabella Kahhale, Sarah L. H. Kamhout, Arielle S. Keller, Harmandeep Singh Khera, Gregory Kiar, Peter Alexander Kirk, Simon H. Kohl, Stephanie A. Korenic, Cole Korponay, Alyssa K. Kozlowski, Nevena Kraljevic, Alberto Lazari, Mackenzie J. Leavitt, Zhaolong Li, Giulia Liberati, Elizabeth S. Lorenc, Annabelle Julina Lossin, Leon D. Lotter, David M. Lydon-Staley, Christopher R. Madan, Neville Magielse, Hilary A. Marusak, Julien Mayor, Amanda L. McGowan, Kahini P. Mehta, Steven Lee Meisler, Cleanthis Michael, Mackenzie E. Mitchell, Simon Morand-Beaulieu, Benjamin T. Newman, Jared A. Nielsen, Shane M. O’Mara, Amar Ojha, Adam Omary, Evren Özarslan, Linden Parkes, Madeline Peterson, Adam Robert Pines, Claudia Pisanu, Ryan R. Rich, Ashish K. Sahoo, Amjad Samara, Farah Sayed, Jonathan Thore Schneider, Lindsay S. Shaffer, Ekaterina Shatalina, Sara A. Sims, Skyler Sinclair, Jae W. Song, Griffin Stockton Hogrogian, Ursula A. Tooley, Vaibhav Tripathi, Hamid B. Turker, Sofie Louise Valk, Matthew B. Wall, Cheryl K. Walther, Yuchao Wang, Bertil Wegmann, Thomas Welton, Alex I. Wiesman, Andrew G. Wiesman, Mark Wiesman, Drew E. Winters, Ruiyi Yuan, Sadie J. Zacharek, Chris Zajner, Ilya Zakharov, Gianpaolo Zammarchi, Dale Zhou, Benjamin Zimmerman, Kurt Zoner, Theodore D. Satterthwaite, Ariel Rokem

**Affiliations:** 1 Stanford University, Division of Developmental and Behavioral Pediatrics, Stanford, California 94305 USA; 2 Stanford University, Graduate School of Education, Stanford, California 94305 USA; 3 Lifespan Informatics and Neuroimaging Center (PennLINC), Department of Psychiatry, Perelman School of Medicine, University of Pennsylvania, Philadelphia, Pennsylvania 19104 USA; 4 Penn/CHOP Lifespan Brain Institute, Perelman School of Medicine, Children’s Hospital of Philadelphia Research Institute, Philadelphia, Pennsylvania 19104 USA; 5 Department of Psychiatry, Perelman School of Medicine, University of Pennsylvania, Philadelphia, Pennsylvania 19104 USA; 6 Child Mind Institute, Center for the Developing Brain, New York City, New York 10022 USA; 7 University of Modena and Reggio Emilia, Department of Biomedical, Metabolic and Neural Sciences, 41125 Modena, Italy; 8 Nathan Kline Institute for Psychiatric Research, Center for Biomedical Imaging and Neuromodulation, Orangeburg, New York 10962 USA; 9 Stanford University, Department of Psychiatry and Behavioral Sciences, School of Medicine, Stanford, California 94305 USA; 10 University of Zurich, Department of Child and Adolescent Psychiatry and Psychotherapy, University Hospital of Psychiatry Zurich, Zurich, 8032 Switzerland; 11 Neuroscience Center Zurich, University of Zurich and ETH Zurich, Zurich, 8057 Switzerland; 12 University of Washington, Department of Psychology, Seattle, Washington 98195 USA; 13 University of Washington, eScience Institute, Seattle, Washington 98195 USA; 14 Old Dominion University, Norfolk, VA 23529 USA; 15 Carleton University, Northfield, MN 55057 USA; 16 Southern Methodist University, Dallas, TX 75275 USA; 17 University of California-Davis, Davis, CA 95616 USA; 18 University of Pennsylvania, Philadelphia, PA 19104 USA; 19 Harvard University, Cambridge, MA 2138 USA; 20 University of Toronto, Toronto, ON M5T 2S8 Canada; 21 Duke University, Durham, NC 27708 USA; 22 University of Michigan-Ann Arbor, Ann Arbor, MI 48109 USA; 23 San Diego State University, San Diego, CA 92182 USA; 24 University of California-San Diego, La Jolla, CA 92093 USA; 25 Concordia University, Montréal, QC H4B 1R6 Canada; 26 Katholieke Universiteit Leuven, 3000 Leuven, Belgium; 27 University of Manchester, Manchester, M13 9PL United Kingdom; 28 University of Sydney, Camperdown, NSW 2006 Australia; 29 University Medical Center Utrecht, 3584 CX Utrecht, Netherlands; 30 Polytechnique Montreal, Montréal, QC H3T 1J4 Canada; 31 University of Rochester, Rochester, NY 14627 USA; 32 National Institute of Mental Health, Bethesda, MD 20892 USA; 33 Centre de Recherche de l’Institut Universitaire en Santé Mentale de Montréal, Montréal, QC H1N 3M5 Canada; 34 Linköping university, 581 83 Linköping, Sweden; 35 Monash University, Clayton, VIC 3800 Australia; 36 Temple University, Philadelphia, PA 19122 USA; 37 University of Greenwich, London, SE10 9LS United Kingdom; 38 Indiana University-Bloomington, Bloomington, IN 47405 USA; 39 Georgia State University, Atlanta, GA 30303 USA; 40 University College London, London, WC1E 6BT United Kingdom; 41 Imperial College London, London, SW7 2BX United Kingdom; 42 Brigham and Women’s Hospital, Boston, MA 02115 USA; 43 University of North Carolina at Chapel Hill, Chapel Hill, NC 27599 USA; 44 University of Concepción, Concepción, Bio Bio Chile; 45 Universitätsmedizin der Johannes Gutenberg-Universität Mainz, 55131 Mainz, Germany; 46 University of Maryland-College Park, College Park, MD 20742 USA; 47 Philipps-Universität Marburg, Marburg, 35037 Germany; 48 Columbia University, New York, NY 10027 USA; 49 McGill University, Montreal, Quebec H3A 0G4 Canada; 50 University of Pittsburgh, Pittsburgh, PA 15260 USA; 51 University of Montréal, Montreal, Quebec, H3T 1J4 Canada; 52 Universidade de São Paulo, Ribeirão Preto, Brazil; 53 Heinrich-Heine University Dusseldorf, 40225 Düsseldorf, Germany; 54 Yale University, New Haven, CT 6520 USA; 55 University of Chicago, Chicago, IL 60637 USA; 56 Brigham Young University, Provo, UT 84602 USA; 57 Child Mind Institute, New York, NY 10022 USA; 58 Institute of Neurosciences and Medicine, Forschungszentrum Jülich, 52425 Jülich, Germany; 59 McLean Hospital, Belmont, MA 02478 USA; 60 University of Oxford, Oxford, OX1 2JD United Kingdom; 61 Auburn University, Auburn, AL 36849 USA; 62 Washington University in St Louis, Saint Louis, MO 63130 USA; 63 Université catholique de Louvain, 1348 Ottignies-Louvain-la-Neuve, Belgium; 64 University of Texas at Austin, Austin, TX 78705 USA; 65 University of Nottingham, Nottingham, NG7 2RD United Kingdom; 66 Wayne State University, Detroit, MI 48202 USA; 67 University of Oslo, 0315 Oslo, Norway; 68 University of Michigan, Ann Arbor, MI 48109 USA; 69 University of Virginia, Charlottesville, VA 22903 USA; 70 Trinity College Dublin, Dublin, 2 Ireland; 71 Linköping University, 581 83 Linköping, Sweden; 72 Stanford University, Stanford, CA 94305 USA; 73 University of Cagliari, 09124 Cagliari, CA Italy; 74 University of Florida, Gainesville, FL 32611 USA; 75 George Mason University, Fairfax, VA 22030 USA; 76 University of Alabama at Birmingham, Birmingham, AL 35294 USA; 77 Boston University, Boston, MA 2215 USA; 78 Cornell University, Ithaca, NY 14853 USA; 79 Max Planck Institute for Human Cognitive and Brain Sciences, 04103 Leipzig, Germany; 80 National Neuroscience Institute, Singapore, 308433 Singapore; 81 University of Colorado School of Medicine, Aurora, CO 80045 USA; 82 Massachusetts Institute of Technology, Cambridge, MA 02139 USA; 83 Western Unviersity, London, ON N6A 3K7 Canada; 84 Psychological Institute of Russian Academy of Education, Moscow, 129366 Russia; 85 University of Illinois Urbana-Champaign, Champaign, IL 61820 USA

**Keywords:** Cognitive neuroscience, Computational neuroscience

Correction to: *Scientific Data* 10.1038/s41597-022-01695-7, published online 12 October 2022

In this article the affiliation details for Bárbara Avelar-Pereira were incorrectly given as “University of Zurich, Department of Child and Adolescent Psychiatry and Psychotherapy, University Hospital of Psychiatry Zurich, Zurich, 8032, Switzerland” but should have been “Stanford University, Department of Psychiatry and Behavioral Sciences, School of Medicine, Stanford, California, 94305, USA”.

The original article has been corrected.

